# Desensitization of TRPA1 by dimethyl itaconate attenuates acute and chronic pain in mice

**DOI:** 10.3389/fphar.2025.1671461

**Published:** 2025-09-29

**Authors:** Sen Xu, Jia-Yue Zhao, Jun-Yi Ma, Xia-Lin Cui, Jia-Hui Lin, Shi-Yu Sun, Si-Jia Liu, Guo-Kun Zhou, Jiang-Tao Zhang, Peipei Kang, Tong Liu

**Affiliations:** ^1^ Institute of Pain Medicine and Special Environmental Medicine, Nantong University, Nantong, China; ^2^ Department of Anesthesiology, Affiliated Tumor Hospital of Nantong University, Nantong, China

**Keywords:** dimethyl itaconate, TRPA1, desensitization, pain, dorsal root ganglion

## Abstract

**Introduction:**

Chronic pain remains a significant clinical challenge due to the limited efficacy of current analgesics. Dimethyl itaconate (DMI), a cell-permeable derivative of itaconate with known anti-inflammatory and immunomodulatory properties, has recently shown promise in alleviating pain. However, the mechanisms by which DMI modulates acute and chronic pain remain unclear.

**Methods:**

Calcium imaging was employed to assess the activation and desensitization effects of DMI on TRPA1 in hTRPA1-HEK293T cells and DRG neurons. Molecular docking analysis was conducted to evaluate the potential covalent binding sites between DMI and TRPA1. Behavioral assays were used to establish acute and chronic pain models in mice and to examine the analgesic effects of DMI in these models.

**Results:**

In the present study, we found that DMI directly activates and desensitizes the transient receptor potential ankyrin 1 (TRPA1) channel, a critical calcium-permeable ion channel implicated in various pain states. Molecular docking analysis and functional assays using calcium imaging revealed possible covalent interactions between DMI and key TRPA1 residue (cysteine 621). To further explore the possible therapeutic effects of DMI for chronic pain, we investigated the possible analgesic effects of DMI in multiple chronic pain mouse models. Single intraplantar injection of DMI induced transient mechanical hypersensitivity in a dose-dependent manner, while repeated injection of DMI failed to induce pain responses in mice. Furthermore, repeated intraperitoneal administration of DMI alleviated pain-related behaviors in a variety of acute pain models, including allyl isothiocyanate (AITC)- and formalin-induced acute inflammatory pain. Moreover, DMI alleviated pain-related behaviors in chronic pain models, including dextran sulfate sodium (DSS)- induced colitis, complete Freund’s adjuvant (CFA)-induced inflammatory pain, oxaliplatin-induced neuropathic pain, and bone cancer pain in mice. Finally, the anti-hyperalgesia effects of DMI on CFA-induced inflammatory pain was abolished in TRPA1 knockout mice.

**Discussion:**

Together, our findings demonstrate that DMI acts as a novel TRPA1 agonist for attenuating acute and chronic pain, possible through TRPA1 desensitization. Thus, DMI may be further developed as a potential therapeutic strategy for the treatment of acute and chronic pain.

## 1 Introduction

Pain is defined by the International Association for the Study of Pain (IASP) (Raja et al., 2020) as “an unpleasant sensory and emotional experience associated with, or resembling that associated with, actual or potential tissue damage.” Most studies consider pain in an acute/chronic dichotomy. Acute pain is a protective response to an injury which informs people of imminent danger. Chronic pain is defined as pain that persists or reoccurs for more than 3 months and causes immense suffering (Treede et al., 2019; Jiang et al., 2020; Lu et al., 2021; Ma et al., 2021), as a consequence of nerve injury, tissue damage, and cancer invasion or treatment, can last for weeks, months or even years, and is detrimental and maladaptive to human bodies (Petrosky et al., 2018; Corder et al., 2019). Chronic pain affects an estimated 20% of people worldwide and account for 15%–20% of physician visits. When felt acutely, pain evokes an innate nociceptive response that aids the body in avoiding further harm. While this response may be evolutionarily useful, numerous conditions and diseases can lead to the generation of pathological chronic pain. However, therapies for pain have not had significant improvement for several decades. The current main therapies including non-steroidal anti-inflammatory drugs (NSAIDs) and opioids are inadequate for the alleviation of chronic pain. Thus, it is of importance to get a better understanding of pain mechanisms to design new therapeutic strategies and analgesics.

Itaconate, a metabolite generated by immune cell activation via the tricarboxylic acid cycle (TCA cycle), exhibits anti-inflammatory and immunomodulatory properties (Lampropoulou et al., 2016). Dimethyl itaconate (DMI), an itaconate derivative capable of permeating cells, has also demonstrated promising efficacy in both anti-inflammatory and antibacterial aspects (Gu et al., 2020). Its structure consists of a central α,β-unsaturated dicarboxylate backbone (C=C-COO-) with two esterified methyl groups attached to the carboxyl moieties. The molecule features a conjugated double bond adjacent to one of the ester groups, which contributes to its electrophilic reactivity. DMI is relatively polar due to the ester functionalities, yet the conjugated unsaturated system allows potential Michael addition reactions with nucleophiles (Mills et al., 2018). Due to its anti-inflammatory and immunomodulatory properties, itaconate has recently become a research focus in the immunometabolism field. Growing evidence indicates that itaconate derivatives have therapeutic effects in idiopathic pulmonary fibrosis (Ogger et al., 2020), ischaemia-reperfusion injury (Zhang et al., 2019), abdominal aortic aneurysm (Song et al., 2020), sepsis (Zhang et al., 2021), multiple sclerosis (Kuo et al., 2020) and endometritis (He et al., 2025). Previously, researchers have examined the analgesic effects of itaconate administered intraperitoneally and spinally in male and female rats with chronic nerve injury (Sun et al., 2022). Recent studies have also found that DMI alleviated chronic pain symptoms in spinal nerve ligation (SNL) (Ren et al., 2022) and CFA-induced inflammatory pain models (Lin et al., 2022; Ren et al., 2022). Besides, administering DMI can alleviate pain in rats during formalin test (Abbaszadeh et al., 2024; Rahimi et al., 2024). These studies all indicate that DMI plays a crucial role in pain regulation. However, its regulatory mechanism for acute and chronic pain remains unknown.

Transient receptor potential (TRP) channels, which were first discovered in 1969 (Cosens and Manning, 1969), are multimodal ion channels that act as sensors of chemically toxic and physical stimuli (Chen et al., 2020). These channels are widely distributed in various tissues and play a variety of roles (Hof et al., 2019; Zhang et al., 2023). TRP channels are nonselective cation channels that can be activated by various stimuli, including changes in temperature, mechanical forces, osmotic pressure, and both endogenous and exogenous chemical compounds. These channels play a crucial role in the development of pathological processes such as inflammation and pain reduction (Moore et al., 2018; Liang et al., 2023). Despite having been studied for a long time, the significance of TRP channels remains unclear. Transient receptor potential ankyrin 1 (TRPA1) is a calcium-permeable non-selective homotetrameric cation channel expressed in a subset of primary sensory neurons of the dorsal root, trigeminal, and nodose ganglia where it plays a role in many pain-associated diseases (da Costa et al., 2010; Zhang, 2015). Although TRPA1 is an attractive therapeutic target for pain, its underlying mechanisms in pain modulation remain to be further elucidated.

In this study, we demonstrated that DMI activates TRPA1 by using calcium imaging, and molecular docking analyses revealed a possible covalent interaction between DMI and cysteine 621 (Cys621) of TRPA1, indicating a specific molecular binding site. Mutation of the Cys621 residue significantly attenuated the ability of DMI to activate TRPA1. Further calcium imaging results showed that repeated perfusion of DMI at the same concentration induced TRPA1 desensitization. To explore the physiological relevance of this desensitization, we performed behavioral analyses. Intraplantar injection of DMI elicited mechanical hypersensitivity in a dose-dependent manner, while repeated administration reduced the nocifensive responses. Moreover, repeated intraperitoneal administration of DMI alleviated pain behaviors, motor dysfunction, and anxiety-like phenotypes across various acute and chronic pain models, including DSS-induced colitis, CFA-induced inflammatory pain, AITC- and formalin-induced nociceptive pain, oxaliplatin-induced neuropathic pain, and a bone cancer pain model. Finally, the anti-hyperalgesia effects of DMI on CFA-induced inflammatory pain was abolished in TRPA1 knockout mice. Together, these findings suggest that targeting TRPA1 desensitization induced by DMI may represent a novel therapeutic strategy for pain management.

## 2 Materials and methods

### 2.1 Animals

Male C57BL/6 mice (6–8 weeks old) were obtained from the Shanghai SLAC Laboratory Animal Co., Ltd (Shanghai, China). Male TRPA1^−/−^ mice were purchased from the Jackson Laboratory (Bar Harbor, ME, United States). All animals were housed under a 12-h light/dark cycle with food and water available *ad libitum*. The room was maintained at 22 °C ± 2 °C with 40%–60% humidity. All animal experiment protocols in this study were reviewed and approved by the Animal Care and Use Committee of Nantong University (Reference: S20210305-025) and were conducted in accordance with the National Institutes of Health Guide for the Care and Use of Laboratory Animals. For the euthanasia methods, mice were placed in a plexiglass chamber with 5% isoflurane for 5 min, and decapitated when fully sedated, as measured by a lack of active paw reflex.

### 2.2 Drugs and reagents

Dimethyl itaconate, HC-030031 (a selective TRPA1 inhibitor), capsaicin (a TRPV1 agonist), dextran sulfate sodium salt (DSS), and carvacrol were purchased from MedChemExpress (MCE, Shanghai, China). Allyl isothiocyanate (AITC, a TRPA1 agonist) and formalin were obtained from Sigma-Aldrich (Shanghai, China). Complete Freund’s adjuvant (CFA) was purchased from Chondrex, Inc. (Woodinville, WA, United States). Oxaliplatin and acetone were obtained from Sinopharm Chemical Reagent Co., Ltd. (Shanghai, China).

### 2.3 Mouse pain model establishment

#### 2.3.1 Formalin-induced nociception pain in mice

The formalin test is used to assess nociception-related responses. It has an acute phase (Phase I) and a prolonged and continuous response (Phase II). Phase I occurs 5 min after formalin administration and results in acute peripheral pain. This pain is likely caused by the direct activation of nociceptors. The interphase follows, during which there is a period of analgesia. Finally, Phase II begins due to continuous inflammatory input and central pain sensitization. Each mouse was placed in the formalin test chamber for 20 min to adapt to the new environment. Individual mice were restrained, after the administration of formalin (2.5% in saline, 10 µL) in the hind paw, the animal’s response (lifting, biting or licking the injected hind paw) to the nociceptive stimulus was recorded for 60 min.

#### 2.3.2 AITC-induced nociception pain in mice

Each mouse was placed in the test chamber for 20 min to adapt to the new environment. After the administration of 20 µg of AITC in the hind paw, the mice were placed in a wire mesh cage immediately and the animal’s response durations (lifting, biting or licking the injected hind paw) to the nociceptive stimulus was recorded for 10 min.

#### 2.3.3 DSS-induced colitis in mice

Seven- to eight-week-old sex-matched co-housed littermates were administered 3% DSS in their drinking water *ad libitum* for seven consecutive days. Survival and clinical parameters, such as weight loss, rectal bleeding and diarrhoea were monitored daily. Weight loss was scored as follows: 0, none; 1, 1%–5%; 2, 5%–10%; 3, 10%–20%; and 4, >20%. Stool consistency was scored as follows: 0, well-formed pellets; 1, semisolid and not adhered to the anus; 2, semiformed and adhered to the anus; and 4, liquid stools and adhered to the anus and diarrhea. The bleeding score was assigned as follows: 0, no blood in stool; 1, light and faint; 2, clear and visible; and four gross rectal bleeding. The sum of the weight loss, stool consistency and bleeding scores (disease activity index, DAI) was calculated to assess the overall disease severity of each mouse.

#### 2.3.4 CFA-induced inflammatory pain in mice

An inflammatory pain model was established by the injection of CFA (10 μL) into the plantar surface of the left hind paw (Sun et al., 2012). As an internal control, the mice were treated with the same volume of sterile saline injections. Behavioural measurements were performed on day 1, 3, 5, 7 and 9.

#### 2.3.5 Oxaliplatin-induced neuropathic pain in mice

In mice treated with oxaliplatin (2.4 mg/kg), the drug was administered intraperitoneally for 2 weeks (10 total injections: once daily for five consecutive days, followed by 2 days off, and then a second round of once-daily injections for another 5 days). Oxaliplatin was dissolved in a 5% glucose solution. Control animals received an equivalent volume of vehicle. Mechanical sensitivity and acetone test were performed on day 3, 6, 9, 12, 15, 18 and 21.

#### 2.3.6 LLC-Luc tumor cells-induced bone cancer pain in mice

The murine cell lines lewis lung carcinoma luciferase (LLC-Luc) were lightly digested using 0.05% trypsin, followed by centrifugation to remove poorly digested cell clusters. Cells were then resuspended in PBS at a concentration of 1 × 10^8^ cells/mL. The inoculation was performed as previously described (Wang et al., 2021). Briefly, mice were anesthetized with 4% isoflurane and the left leg was shaved and the skin disinfected with 10% povidone-iodine and 75% ethanol. A superficial incision (0.5–1 cm) was made near the knee joint, exposing the patellar ligament. A new 25-gauge needle was inserted at the site of the intercondylar notch of the left femur into the femoral cavity, which was then replaced with a 10 µL microinjection syringe containing a 2 µL suspension of tumor cells (2 × 10^5^) followed by 2 µL absorbable gelatin sponge solution to seal the injection site. The syringe contents were slowly injected into the femoral cavity over a 2-min interval. To prevent further leakage of tumor cells outside of the bone cavity, the outer injection site was sealed with silicone adhesive. Animals with surgery related movement dysfunction or with outside bone tumor injection were excluded from the study.

### 2.4 Behavioral testing

#### 2.4.1 von Frey test

Briefly, mice were placed individually in a plexiglass chamber designed for the evaluation of mechanical thresholds and were habituated to the room temperature for at least 1 h before the test. Then, a series of von Frey hairs in logarithmic increments of force (0.008, 0.02, 0.04, 0.07, 0.16, 0.4, 0.6, 1, 1.4 g) were used to stimulate the injected hind paw. The response was considered positive when the mouse strongly withdrew or flinching/licking/biting of the paw. The von Frey hairs were applied with sufficient force to cause slight buckling and held for approximately 2–4 s. Absence of response after 5 s led to the use of a filament with increased weight, whereas a positive response led to the use of a weaker (i.e., lighter) filament. Ten measurements were collected for each mouse with at least a 5 min interval between two consecutive applications or until five consecutive positive or negative responses occurred. The 50% mechanical withdrawal threshold (expressed in g) response was then calculated from these scores. And the paw withdrawal frequencies (PWFs) in response to mechanical stimuli were measured according to the frequency of the withdrawal response. Mechanical nociceptive threshold was determined before (basal level) and after different treatments.

For the abdominal mechanical pain threshold test, mice were individually placed in transparent Plexiglas chambers on an elevated wire mesh platform and allowed to acclimate for at least 1 h. Abdominal mechanical thresholds were assessed using a series of von Frey filaments (0.008–1.4 g) applied perpendicularly to the abdominal surface until slight bending occurred and held for 2–4 s. A positive response (abdominal withdrawal, licking, or escape behavior) prompted the use of the next lower-force filament, whereas a negative response led to the application of the next higher-force filament. Ten measurements were obtained per mouse with ≥5 min intervals between stimulations or until five consecutive identical responses were recorded. The 50% withdrawal threshold (g) was calculated using the up-down method. Baseline thresholds were measured before treatment and re-assessed at designated time points after interventions.

#### 2.4.2 Hargreaves test

Each mouse was placed in a transparent, square, bottomless plexiglass box on a glass plate. After an acclimation period of 30 min, the thermal paw withdrawal latency period to infrared heat (cutoff of 20 s) was measured three times with a Hargreaves apparatus at 10-min intervals. The average thermal paw withdrawal latency of three trials was calculated for analysis.

#### 2.4.3 Acetone test

The acetone test was performed according to the method described by previous publications (Xie et al., 2016). Mice were placed to the same setting described above for the von Frey test and allowed to habituate for 45 min prior to testing. Fifty microliters of acetone were applied to the center of the ventral side of the hind paw and responses were observed. In the first 20 s following acetone application, if the mouse did not withdraw, flick or stamp of the paw then 0 points were recorded for the trial. However, if within this 20 s period the animal responded to acetone, then the animal’s response was assessed for an additional 20 s. Responses to acetone were graded according to the following four-point scale: 0, no response; 1, quick withdrawal, flick or stamp of the paw; 2, prolonged withdrawal or repeated flicking of the paw; 3, repeated flicking of the paw with licking directed at the paw. Acetone test was applied alternately three times to each paw and the responses scored categorically.

#### 2.4.4 Rotarod test

The locomotor activity of mice was tested with the accelerating rotarod test. Mice were placed on a rotarod apparatus, set to accelerate from 4 to 40 rpm over 300 s and the time to the end of each trial was recorded. The trial ended when the animal either fell off the rotarod, completed two full rotations when clinging on to the rod or when it ran for 300 s. Animals first underwent training for three consecutive days, consisting of 10 rotarod trials separated by a 3-min rest period each day. This allowed the animals to learn the task and reach a plateau in their performance by the third day of training. After completion of the training, animals were tested on the rotarod.

#### 2.4.5 Open filed test

To assess the activity capacity and anxiety-like behavior of mice, a 500 × 500 × 350 mm open field were set as 16 equally squares to record the animal trace. The environment should be quiet and the box should be cleaned with 70% ethanol just like before. Before the test, mice were taken to the room to adapt to the new environment for nearly 1 h. After placing the mouse in the open field facing the corner of the box, recording of the animal activity was started immediately and monitored for 5 min. Then, the mice were put back into the feeding cages. A less anxious mouse tends to be interested in the central area and shows a desire to explore. While mice with high anxiety tend to walk against the walls of the box or hide in corners.

#### 2.4.6 Elevated plus maze (EPM) test

An EPM was made of ABS resin and consisted of a central square (6 × 6 cm) and four arms (30 cm long × 6 cm wide, two open arms with no railing and two closed arms enclosed by a transverse wall 15 cm in height). The maze was elevated 50 cm from the floor. In a recording session, the mice were put in the central area and made them face the open arm to explore the maze for 5 min. The process was recorded by a video camera and the maze was cleaned with 70% ethanol to remove residual odors before each test. The mice with higher anxiety would visit the open arm less often and stay there for a shorter time.

### 2.5 Acutely isolated dorsal root ganglia neurons

Dorsal root ganglion neurons were dissociated and prepared from mice (6–8 weeks-of-age) using a similar protocol as previously described (Wu et al., 2021; Zhou et al., 2023). In brief, mice were decapitated after anesthesia with isoflurane. Lumbar DRGs were rapidly removed and placed in ice-cold oxygenated balanced D-Hank’s solution (Solarbio, Beijing, China). The DRGs were then digested in Dulbecco’s Modified Eagle Medium (DMEM, Gibco, Grand Island, NY, United States) containing collagenase D (0.6 U/mL; Roche, Mannheim, Germany) and dispase II (3.0 U/mL; Roche, Mannheim, Germany) for 35–40 min at 37 °C. The ganglia were then triturated with fire-polished Pasteur pipettes. The dispersed cells were resuspended in F12 (Biological Industries, Beit HaEmek, Israel) medium supplemented with 10% FBS (Gibco, Waltham, MA, United States) and 1% penicillin/streptomycin (Biosharp, Hefei, China) and plated on coverslips coated with Poly-D-lysine (BBI Lifescience, Shanghai, China). Cell cultures were maintained in regular 95% air and 5% CO_2_ at 37 °C in an incubator.

### 2.6 Cell culture

The human embryonic kidney epithelial cell line HEK-293T and the Lewis lung carcinoma luciferase cell line (LLC-Luc) were propagated in Dulbecco’s modified Eagle’s medium (DMEM, Gibco, United States) supplemented with 10% fetal bovine serum (FBS, Gibco, United States) and 1% penicillin/streptomycin (Solarbio, China). All cells were cultured in culture dishes and maintained in regular 95% air and 5% CO_2_ at 37 °C in an incubator. HEK293T cells were transfected with 1 µg of a human TRPA1 (hTRPA1) cDNA or a human TRPV1 (hTRPV1) cDNA by using Lipofectamine 3000 (Invitrogen, Carlsbad, CA, United States). And the LLC-Luc cells were used to construct the bone cancer pain model in mice.

### 2.7 Calcium imaging

Single cell intracellular calcium imaging was measured in untransfected and in hTRPA1-HEK293T, hTRPV1-HEK293T cells, or in primary cultured DRG neurons. Primary cultured DRG neurons and HEK293T cells were loaded with 1 μg/mL Fura-2 a.m. (1:1,000, Thermo Fisher) and 0.01% F-127 (w/v; Invitrogen) for 30 min in a 37 °C incubator with 5% CO_2_. Images were captured via a CCD camera (PCO, Germany) integrated with an inverted microscope (Nikon, Japan). The excitation of Fura-2 was facilitated by an alternating light source (PTI, United States) emitting wavelengths of 340 and 380 nm. In chambers equipped with a custom four-channel perfusion valve control system, cells were infused with calcium imaging buffer (CIB) (130 mM NaCl, 5.6 mM KCl, 2.6 mM CaCl_2_, 1.2 mM MgCl_2_, 10 mM HEPES, and 5.6 mM d-glucose at pH 7.4) to baseline, then infused the test solution, and finally infused with CIB to baseline. Changes in calcium levels were expressed as the relative change in the 340/380 ratio from baseline. The normalized percentage of response amplitude was calculated relative to the maximal agonist- or 56 mM KCl-evoked peak response under each experimental condition.

### 2.8 Molecular docking

The molecular docking analysis was performed using Discovery Studio Software platform in Shanghai University. The 3D structure of DMI was obtained from PubChem (CID: 69240), the 3D structures of human TRPA1 was available in UniProt (ID: 6V9W), and they were used for CDOCK (A small molecule-protein docking algorithm) analysis, which is based on energy minimization and simulated annealing techniques. The study selected a suitable grid size and center that covers all possible binding regions in the TRPA1, and adjusted Exhaustiveness to a high value of 32 to increase search space and precision and reduce the probability of false positives and false negatives. The TYL06 force field was used for the molecular docking study, which is a molecular force field specifically designed for protein-small molecule interactions. Compared with other force fields, TYL06 force field adopts a second-order moment approximation method in handling charge distribution and considers the dynamic process of hydrogen bond formation, which can more accurately calculate the charge interaction between drug molecules and receptors and describe the hydrogen bond interaction between drug molecules and receptors. The study adjusted the number of optimal molecular docking conformations generated by the search algorithm to 10, which broadened the search space and improved the probability of finding the optimal solution. The study employed a global optimization search algorithm based on genetic algorithms, which often produces multiple optimal solutions with very subtle differences, and represents different poses or orientations that the ligand can adopt within the binding site. Then the covalent docking studies were performed on the structurally refined and energy minimized structure of DMI-hTRPA1. The calculations were performed selecting CYS621 as the covalently modified residues and the DMI as the ligand moiety covalently bound to the residues. 10 different ligand binding orientations were evaluated, and the top-scored disposition was considered for further analyses.

### 2.9 Histology

Following 7 days of 3% DSS administration, mice were killed and sections of caecum, transverse colon, and descending colon were removed and placed in paraformaldehyde. Specimens were then paraffin embedded and subsequently cut into 5 μM sections. Sections were stained with haematoxylin and eosin (H&E). The histological activity index (HAI) was used to grade the severity of intestinal inflammation in accordance with a previously publication (Ye et al., 2022).

### 2.10 Statistical analysis

Results are presented as the mean ± standard error of the mean SEM from a minimum of three biological replicates, as specified in the figure legends. A difference between two treatment groups was analyzed using the unpaired, two-tailed Student’s t-test. One-way ANOVA or two-way ANOVA with a Bonferroni *post hoc* test was used for comparisons of >2 treatment groups/treatment conditions. All statistical analyses were performed using GraphPad Prism 8.0.2 (GraphPad Software Inc., La Jolla, CA, United States). A *P* value < 0.05 was set as the threshold for significance.

## 3 Results

### 3.1 DMI activates exogenously-expressed TRPA1 in HEK293T cells

**FIGURE 1:**
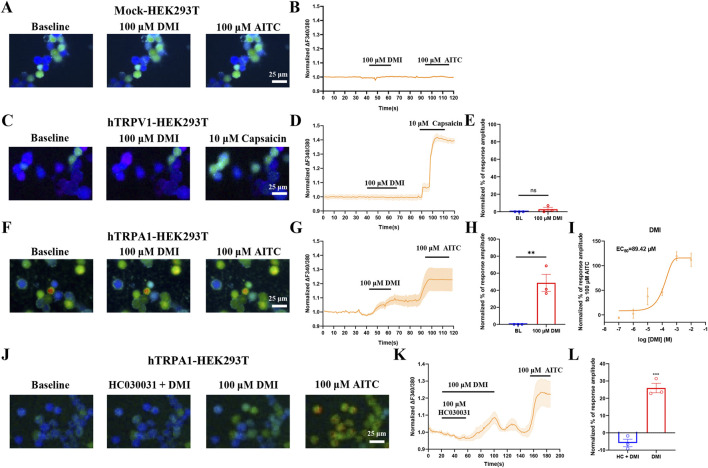
DMI activates exogenously-expressed TRPA1 in HEK293T cells. **(A**,**C**,**F)** Representative images of mock-HEK293T, hTRPA1-HEK293T and hTRPV1-HEK293T cells in response to 100 μM DMI, 100 μM AITC or 10 μM capsaicin. Scale bar: 25 μm. **(B**,**D**,**G)** Representative traces of mock-HEK293T, hTRPV1-HEK293T and hTRPA1-HEK293T cells in response to 100 μM DMI, 100 μM AITC or 10 μM capsaicin. **(E**,**H)** Normalized percentage peak response of mock-HEK293T, hTRPV1-HEK293T and hTRPA1-HEK293T cells in response to 100 μM DMI relative to 100 μM AITC or 10 μM capsaicin (^**^ *p* < 0.01 vs. Baseline, n = 3 replicates per treatment, unpaired, two-tailed Student’s t-test). **(I)** Quantification of peak calcium responses (ΔF/F_0_) normalized to baseline, and the dose-response curve fitted by nonlinear regression showing a calculated EC_50_ value of DMI-induced TRPA1 activation in hTRPA1-HEK293T cells (n = 3 replicates per treatment, nonlinear regression). **(J)** Representative images of hTRPA1-HEK293T cells in response to 100 μM DMI, 100 μM HC030031 and 100 μM AITC. Scale bar: 25 μm. **(K)** Representative traces of hTRPA1-HEK293T cells in response to 100 μM DMI, 100 μM HC030031 and 100 μM AITC. **(L)** Normalized percentage peak response of hTRPA1-HEK293T cells to 100 μM DMI and 100 μM HC030031 relative to 100 μM AITC (^***^ *p* < 0.001 vs. Baseline, n = 3 replicates per treatment, unpaired, two-tailed Student’s t-test).

TRPA1 and TRPV1 are two TRP channels known to play crucial roles in the detection and transmission of itch and pain (Patapoutian et al., 2009; Sun and Dong, 2016). TRPA1 is a non-selective cation channel expressed at sensory nerve terminals and is highly permeable to Ca^2+^. To determine the specific TRP channel involved in the effect of dimethyl itaconate (DMI), we transiently transfected HEK293T cells with human TRPA1 or TRPV1 plasmids and monitored intracellular calcium signals using live-cell calcium imaging. Perfusion of 100 μM DMI robustly increased calcium influx in hTRPA1-overexpressing HEK293T cells (hTRPA1-HEK293T) (Figures 1F–H), whereas no response was observed in either mock-transfected cells (Mock-HEK293T) (Figures 1A,B) or hTRPV1-overexpressing cells (hTRPV1-HEK293T) (Figures 1C–E). Furthermore, DMI induced a concentration-dependent increase in intracellular calcium in hTRPA1-HEK293T cells. Nonlinear regression analysis revealed an EC_50_ of approximately 89.42 μM for DMI-induced TRPA1 activation (Figure 1I).

To confirm that TRPA1 is the direct target of DMI, we co-applied 100 μM DMI with the selective TRPA1 antagonist HC-030031 (100 μM) to hTRPA1-HEK293T cells. HC-030031 effectively suppressed the DMI-induced calcium signal (Figures 1J–L). Upon washout of the antagonist, subsequent perfusion of DMI restored TRPA1 activation and intracellular calcium elevation (Figures 1J–L). These results demonstrate that TRPA1 specifically mediates DMI-induced calcium influx in HEK293T cells.

### 3.2 DMI induces TRPA1-dependent activation in acute dissociated DRG neurons

**FIGURE 2:**
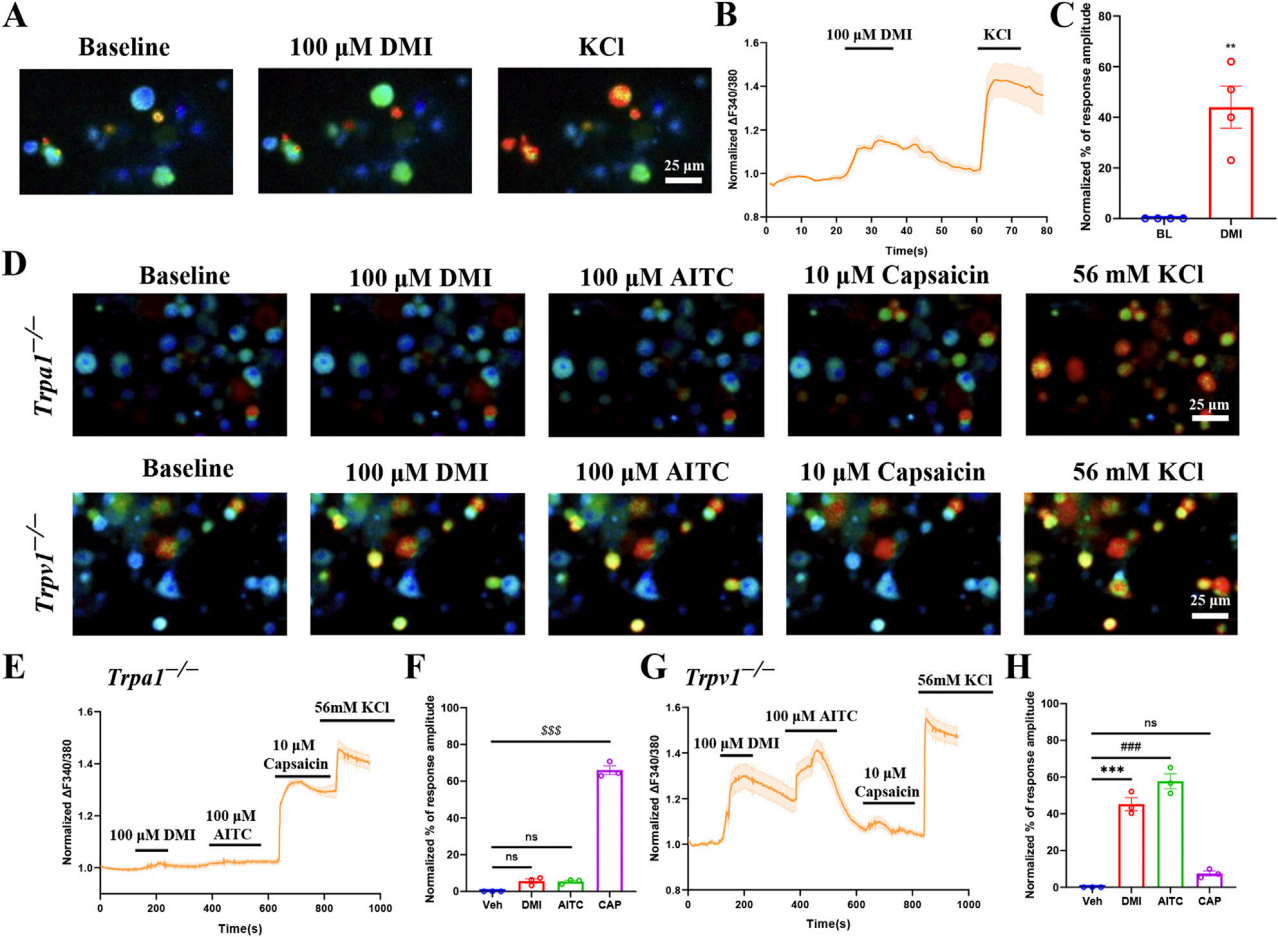
DMI activates TRPA1 in acute dissociated DRG neurons. **(A)** Representative images of DRG neurons responses to 100 μM DMI. Scale bar: 25 μm. **(B)** Representative of DRG neurons in response to 100 μM DMI. **(C)** Normalized percentage peak response of DRG neurons in response to 100 μM DMI (^**^ *p* < 0.01 vs. Baseline, n = 4 replicates per treatment, approximately 200 neurons per group, unpaired, two-tailed Student’s t-test). **(D)** Representative images of DRG neurons from TRPA1^−/−^ and TRPV1^−/−^ mice in response to 100 μM DMI, 100 μM AITC and 10 μM capsaicin. Scale bar: 25 μm. **(E–H)** Representative traces and normalized percentage peak response of DRG neurons in from TRPA1^−/−^ and TRPV1^−/−^ mice in response to 100 μM DMI, 100 μM AITC and 10 μM capsaicin (^***^ *p* < 0.001 vs. Baseline, ^###^ *p* < 0.001 vs. Baseline, ^$$$^ *p* < 0.001 vs. Baseline, n = 3 replicates per treatment, approximately 200 neurons per group, one-way AVOVA following Bonferroni’s test).

To investigate whether DMI also activates endogenous TRPA1, we performed live-cell calcium imaging in primary dorsal root ganglion (DRG) neurons isolated from wild-type mice. We included all neurons that exhibited a detectable response to 56 mM KCl depolarization, neurons that did not respond to KCl were excluded from further analysis, as the lack of a depolarization-induced calcium signal suggests poor viability or compromised membrane excitability. For the analysis, we quantified the representative traces of these responsive neurons and calculated their normalized percentage peak responses relative to the peak amplitude evoked by 56 mM KCl. The results showed that perfusion with DMI rapidly increased intracellular calcium levels, indicating activation of primary sensory neurons (Figures 2A–C).

To further validate the specificity of TRPA1 in mediating DMI-induced responses, we conducted the same experiments using DRG neurons derived from Trpa1^−/−^ and Trpv1^−/−^ mice. Calcium imaging results showed that DMI failed to evoke significant calcium responses in Trpa1^−/−^ DRG neurons (Figures 2D–F), while robust calcium signals were still observed in Trpv1^−/−^ DRG neurons (Figures 2D,G,H). These results confirm that DMI selectively activates endogenous TRPA1, but not TRPV1, in mouse sensory neurons.

### 3.3 DMI interacts with TRPA1 and forms a covalent bond at the Cys621 residue

These results confirm that DMI directly activates the TRPA1 channel. To further explore the molecular mechanism of its action, we employed computational biology approaches to systematically investigate the DMI-TRPA1 interaction. First, the molecular structure of DMI (CID: 69240) was downloaded in standard SMILES format from the PubChem database, and the overall structure used for docking was derived from the cryo-EM model of human TRPA1 in its open conformation (PDB ID: 6V9W), represented as a tetrameric assembly of transmembrane helices. The molecular docking was performed using the CDOCKER algorithm in Discovery Studio 4.1, and the pose with the lowest binding free energy, pose 1 (−18.942 kcal/mol), was selected from 10 candidate conformations for further analysis.

**FIGURE 3:**
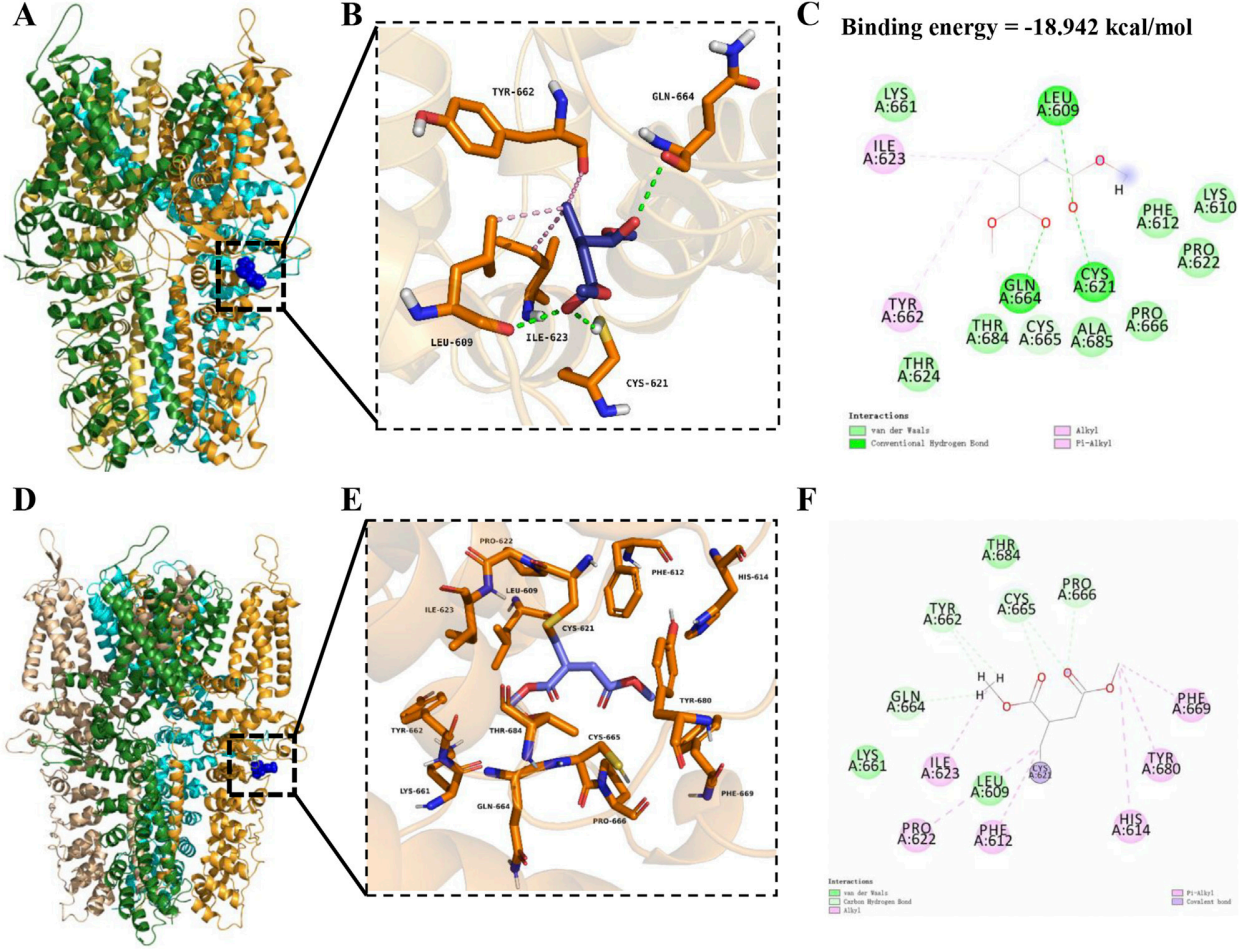
DMI possible covalently binds to TRPA1 at the cysteine residue Cys621. **(A)** Overall cryo-EM structure of human TRPA1 shown as a tetramer, with two adjacent subunits represented in green and orange, highlighting the interfacial binding pocket. DMI is shown in blue stick representation within the pocket. **(B**,**C)** Detailed view of the non-covalent docking pose. Key interacting residues (Leu609, Ile623, Cys621, Tyr662, and Gln664) are displayed in orange sticks, while hydrogen bonds are indicated by green dashed lines. The 2D interaction diagram **(C)** depicts hydrogen bonds (green lines), hydrophobic (alkyl and π–alkyl) contacts (pink lines), and van der Waals interactions (green spheres). **(D)** Overall TRPA1 structure highlighting the covalent docking model, with DMI shown in blue sticks positioned near Cys621. **(E**,**F)** Close-up of the covalent docking site. The α,β-unsaturated ketone group of DMI interacts with the thiol group of Cys621, forming a covalent bond (purple bond highlight), supported by surrounding hydrophobic residues (Tyr662, Phe612, His614) and hydrogen-bonding residues (Gln664, Thr684). The 2D diagram **(F)** summarizes these interactions, with color-coded annotations for hydrogen bonds, hydrophobic contacts, and covalent linkages.

The docking simulations revealed that DMI anchors within the interfacial pocket of the human TRPA1 channel, which is located at the interface between adjacent subunits and involves residues from the transmembrane helices (S5–S6 pore region) as well as residues from the N-terminal ankyrin repeat extension (Figure 3A). In the non-covalent docking model, DMI establishes three key hydrogen bonds with Cys621, Gln664, and Leu609, respectively (Figures 3B,C). Additional hydrophobic contacts were observed between the alkyl chain of DMI and residues Ile623 and Tyr662, and alkyl chain of DMI engages in π–alkyl stacking interactions with Tyr662 (Figures 3B,C). These multimodal interactions collectively form a stable binding interface between DMI and TRPA1, where the polar interactions mediated by the hydrogen bond network and the hydrophobic effects synergistically enhance the binding stability. These findings indicate that DMI effectively activates the TRPA1 channel through a multifaceted cooperative interaction.

Most compounds known to activate TRPA1 can covalently bind cysteine residues (Macpherson et al., 2007; Zhao et al., 2020). To investigate whether DMI forms a stable complex with TRPA1 through covalent interactions, we next utilized a covalent docking module for precise simulation. The calculations were performed selecting CYS621 as the covalently modified residues and the DMI as the ligand moiety covalently bound to the residues. Based on the lowest binding free energy and optimal interaction scoring, pose 1, which showed the most favorable binding mode, was selected from the 10 candidate conformations for further analysis. The covalent docking analysis further demonstrated that the α,β-unsaturated ketone group of DMI is positioned in close proximity to the sulfhydryl group of Cys621, enabling covalent adduction through a Michael addition reaction. The modeling confirmed that Cys621 lies within a reactive pocket of the TRPA1 pore domain, where van der Waals contacts guide the electrophilic moiety into alignment with the cysteine thiol, thereby facilitating irreversible covalent bond formation (Figures 3D–F). This covalent modification results in an ultra-low binding free energy for the DMI-TRPA1 complex. Our study confirms that DMI induces specific covalent modification at the TRPA1 pore, potentially altering the channel’s gating properties through steric hindrance. This provides a novel structural perspective for understanding the covalent activation mechanism of TRPA1.

### 3.4 DMI elicits reduced calcium signals of the C621Y-mutant TRPA1 in HEK293T cells

**FIGURE 4:**
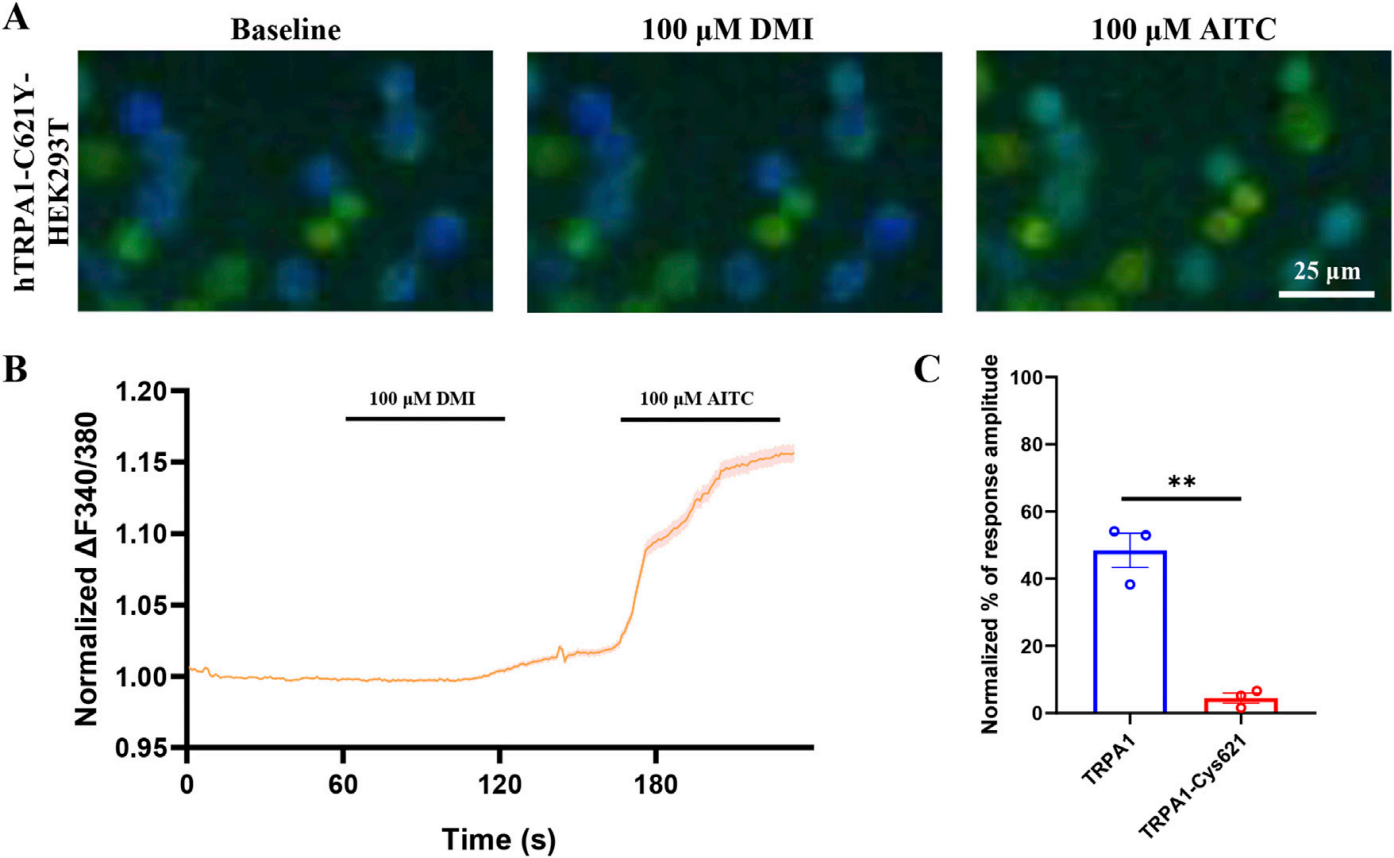
DMI fails to activate C621Y-mutant TRPA1 in HEK293T cells. **(A)** Representative images of hTRPA1-Cys621Y-HEK293T cells in responses to 100 μM DMI. Scale bar: 25 μm. **(B**,**C)** Representative traces and normalized percentage peak response of hTRPA1-Cys621-HEK293T cells in response to 100 μM DMI (^**^ *p* < 0.01 vs. Baseline, n = 3 replicates per treatment, unpaired, two-tailed Student’s t-test).

Previous studies shown that electrophiles activate TRPA1 through covalent modification of cysteine residues within the channel’s cytoplasmic amino terminus (Hinman et al., 2006; Macpherson et al., 2007), Mutation studies have suggested Cys’s (e.g., C414, C621, and C665) are critical to TRPA1 activation by electrophiles (Hinman et al., 2006; Macpherson et al., 2007), although it is not clear whether these Cys’s are involved (directly or indirectly) with binding or activation. The above molecular docking results indicate that DMI is capable of covalently modifying the cysteine residue at position Cys621 of the TRPA1 channel. To determine whether the Cys621 residue is critical for DMI-induced TRPA1 activation, we first constructed a site-directed mutant hTRPA1 plasmid in which Cys621 was substituted by tyrosine. The rationale for this design was to eliminate thiol nucleophilicity and thereby prevent covalent modification by electrophilic ligands, while at the same time preserving side-chain steric bulk and introducing an aromatic group with a polar hydroxyl. This substitution allowed us to test for potential non-covalent contributions (e.g., π-interactions) within the electrophile-sensing pocket. In contrast to classical Ser/Ala substitutions-commonly used to blunt electrophile-evoked activation but significantly reducing side-chain volume-tyrosine was selected to minimize size loss and maintain local packing. The mutant plasmid was transfected into HEK293T cells, and calcium imaging was performed to assess the activation response to DMI. The results showed that mutation of Cys621 significantly attenuated DMI-induced calcium signaling in hTRPA1-expressing HEK293T cells (Figures 4A–C), suggesting that Cys621 is likely a key residue mediating the activation of TRPA1 by DMI.

### 3.5 DMI induces TRPA1 desensitization in primary DRG neurons and hTRPA1-HEK293T cells

**FIGURE 5:**
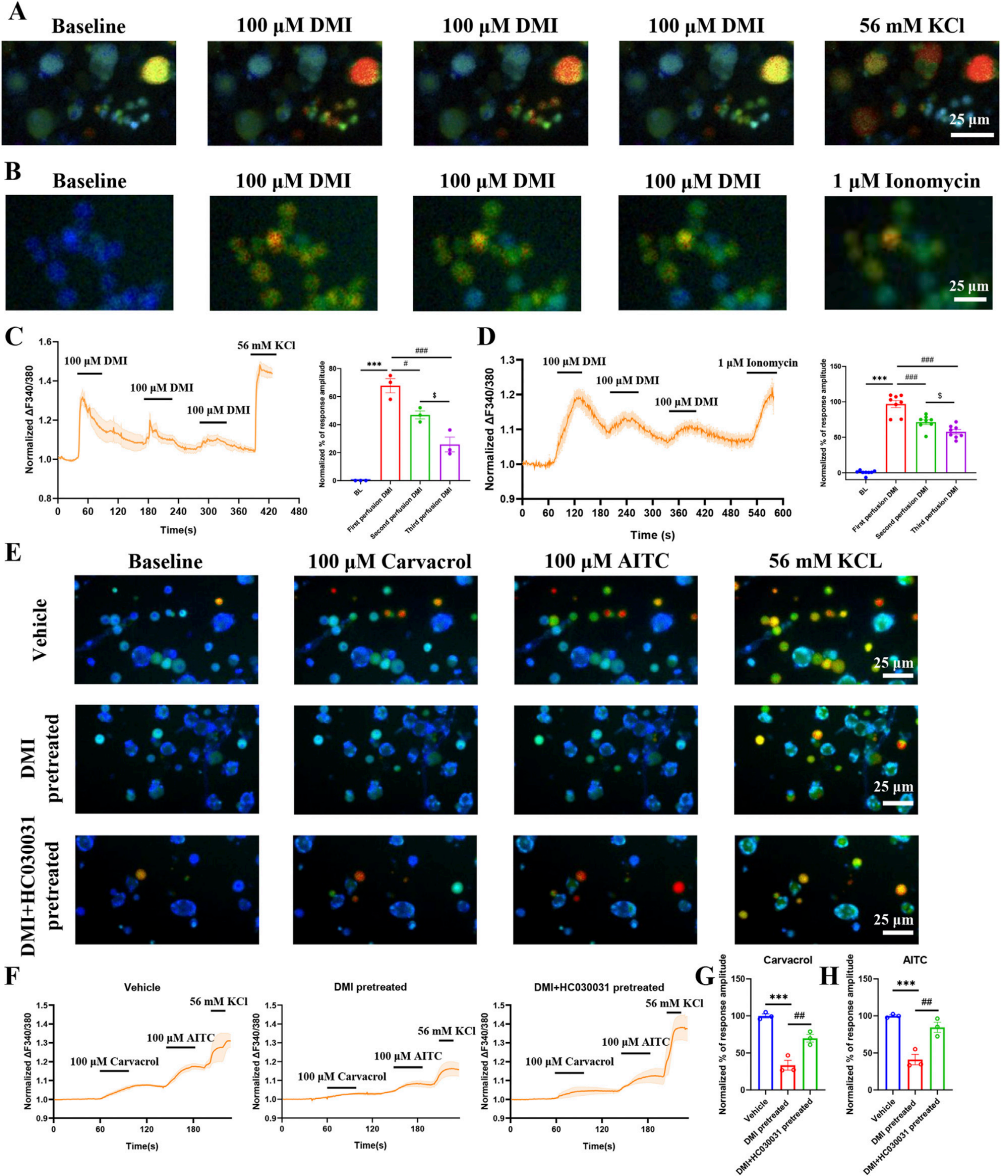
DMI induces TRPA1 desensitization in DRG neurons and hTRPA1- HEK293T cells. **(A)** Representative images of DRG neurons in responses to 100 μM DMI for three times perfusion. Scale bar: 25 μm. **(B)** Representative images of hTRPA1-HEK293T cells in responses to 100 μM DMI for three times perfusion. Scale bar: 25 μm. **(C)** Representative traces and normalized percentage peak response of DRG neurons in response to 100 μM DMI for three times perfusion (^***^ *p* < 0.001 vs. Baseline, ^#^ *p* < 0.05 vs. first perfusion, ^###^ *p* < 0.001 vs. first perfusion, ^$^ *p* < 0.05 vs. second perfusion, n = 3 replicates per treatment, approximately 200 neurons per group, one-way AVOVA following Bonferroni’s test). **(D)** Representative traces and normalized percentage peak response of hTRPA1-HEK293T cells in response to 100 μM DMI for three times perfusion (^***^ *p* < 0.001 vs. Baseline, ^###^ *p* < 0.001 vs. first perfusion, ^$ $^ *p* < 0.01 vs. second perfusion, n = 8 replicates per treatment, one-way AVOVA following Bonferroni’s test). **(E)** Representative images of DRG neurons in response to 100 μM carvacrol and 100 μM AITC after pretreatment with vehicle, DMI (100 μM), or DMI + HC-030031 (100 μM). Scale bar: 25 μm. **(F)** Representative traces of DRG neurons stimulated with 100 μM carvacrol and 100 μM AITC following pretreatment with vehicle, DMI (100 μM), or DMI + HC-030031 (100 μM). **(G)** Normalized percentage peak response of DRG neurons in response to 100 μM carvacrol under the three pretreatment conditions. **(H)** Normalized percentage peak response of DRG neurons in response to 100 μM AITC under the three pretreatment conditions (^***^ *p* < 0.001 vs. Vehicle, ^##^ *p* < 0.01 vs. DMI pretreated, n = 3 replicates per treatment, approximately 200 neurons per group, one-way AVOVA following Bonferroni’s test).

An increasing body of evidence has demonstrated that TRPA1 agonists can serve as an effective strategy to pain management by desensitizing TRPA1 currents (Bressan et al., 2016). To verify whether DMI induces TRPA1 desensitization, we performed functional validation using calcium imaging combined with a cumulative drug application paradigm in acutely dissociated mouse primary dorsal root ganglion (DRG) neurons. The results showed that three consecutive perfusions of 100 μM DMI led to progressively diminished intracellular calcium responses in DRG neurons (Figures 5A,C), suggesting a gradual loss of responsiveness indicative of desensitization.

To further confirm whether this attenuation was mediated by TRPA1 desensitization, the same calcium imaging protocol was applied to hTRPA1-expressing HEK293T (hTRPA1-HEK293T) cells. Consistently, three sequential applications of 100 μM DMI induced a TRPA1-dependent reduction in calcium signaling (Figures 5B,D), further supporting the notion that DMI induces TRPA1-dependent desensitization.

We next investigated whether DMI could also desensitize TRPA1 in response to non-covalent agonists. Using carvacrol, a well-established non-electrophilic TRPA1 activator (Chandrabalan et al., 2019; Mukaiyama et al., 2020), we found that pretreatment with DMI significantly reduced the calcium response to subsequent carvacrol stimulation in DRG neurons (Figures 5E–G). This finding demonstrates that DMI-induced desensitization is not limited to covalent activation but extends to non-covalent ligand responses as well.

Importantly, co-application of the selective TRPA1 antagonist HC-030031 during the DMI pretreatment phase largely reversed the desensitization, as evidenced by restored calcium responses to both carvacrol and AITC (Figures 5G,H). These data indicate that DMI-induced TRPA1 desensitization is channel-specific and pharmacologically reversible, reinforcing the involvement of TRPA1 as a direct molecular target of DMI in sensory neurons.

### 3.6 DMI induces transient mechanical hypersensitivity through TRPA1 activation and repeated intraplantar injection of DMI fails to induce pain in mice

**FIGURE 6:**
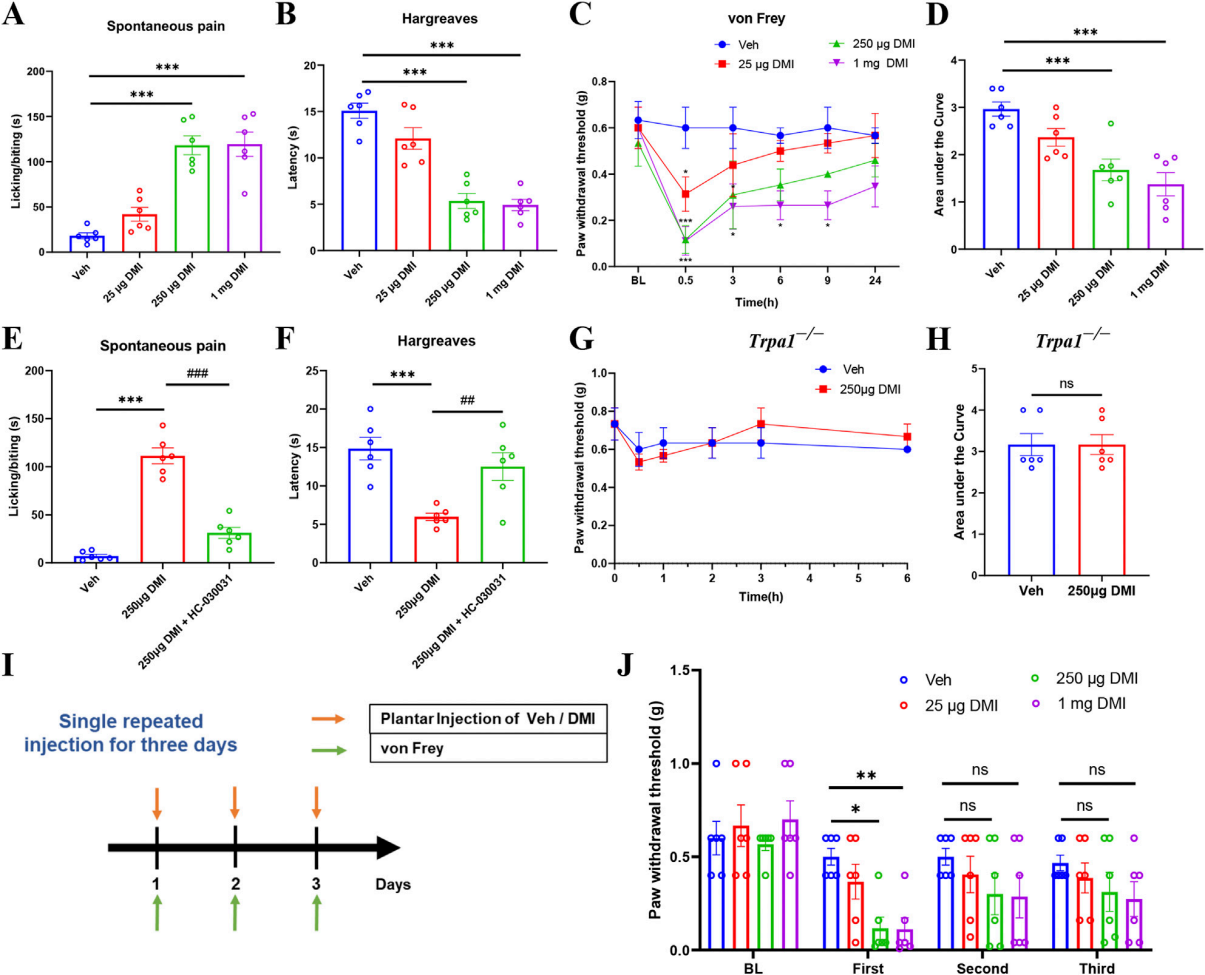
DMI induces dose-dependent transient pain via TRPA1 and causes pain desensitization upon repeated administration. **(A)** Cumulative time of spontaneous pain behaviors within 10 min after plantar injection of different doses of DMI. **(B)** Paw withdrawal latency in the hargreaves test following plantar injection of DMI at various doses. **(C)** Mechanical pain thresholds measured by von Frey test after plantar injection of different doses of DMI. **(D)** Area under the curve (AUC) analysis of mechanical pain thresholds in response to DMI injection (n = 6 per group; ^*^ *P* < 0.05, ^***^ *P* < 0.001 vs. Vehicle; respectively, one-way AVOVA following Bonferroni’s test or two-way AVOVA). **(E)** Quantification of spontaneous pain behaviors within 10 min post-injection. **(F)** Radiant heat withdrawal latency following intraperitoneal injection of the TRPA1 antagonist HC-030031 (100 mg/kg) (n = 6 per group; ^***^ *P* < 0.001 vs. Vehicle; ^##^ *P* < 0.01, ^###^ *P* < 0.001 vs. DMI; one-way AVOVA following Bonferroni’s test). **(G)** Time course of mechanical pain thresholds in Trpa1^−/−^ mice following plantar injection of 250 μg DMI. **(H)** AUC analysis of mechanical pain thresholds in Trpa1^−/−^ mice (n = 6 per group; respectively, unpaired, two-tailed Student’s t-test or two-way AVOVA). **(I)** Schematic diagram of the experimental protocol for repeated DMI injections and mechanical pain assessment. **(J)** Mechanical thresholds after three repeated plantar injections of DMI (n = 6 per group; ^*^ *P* < 0.05 vs. Vehicle; ^**^ *P* < 0.01 vs. Vehicle; two-way AVOVA).

Calcium imaging results confirmed that acute exposure to DMI selectively activates TRPA1, a non-selective cation channel critically involved in nociceptive signaling. To evaluate the behavioral consequences of this activation, we first examined the effects of acutely injected DMI on nociceptive behaviors in naïve mice. Mice received intraplantar injections of DMI at three different doses (25, 250 μg, and 1 mg), followed by quantification of spontaneous pain behaviors within 10 min post-injection. Compared to vehicle-treated controls, DMI induced a dose-dependent increase in spontaneous pain responses, with significant effects observed at 250 μg and 1 mg (Figure 6A). In the radiant heat paw withdrawal assay (cut-off time: 20 s), the paw withdrawal latencies of mice treated with 250 μg and 1 mg DMI were significantly reduced (Figure 6B), indicating thermal hyperalgesia. Mechanical hypersensitivity was assessed at 0.5, 3, 6, 9, and 24 h post-injection. DMI-treated mice exhibited the lowest mechanical thresholds at 0.5 h, which gradually returned toward baseline over time (Figure 6C). The area under the curve (AUC) analysis further supported a dose-dependent mechanical hypersensitivity induced by DMI (Figure 6D). These findings demonstrate that acute intraplantar injection of DMI induces mechanical allodynia and thermal hyperalgesia in naïve mice.

Given the established role of TRPA1 in pain transduction and its potential as a therapeutic target, we next investigated whether TRPA1 mediates DMI-induced pain. Mice were pretreated with the selective TRPA1 antagonist HC-030031 (100 mg/kg, intraperitoneally) prior to intraplantar injection of DMI (250 μg). Behavioral observations revealed that HC-030031 pretreatment significantly reduced DMI-evoked spontaneous pain behaviors within 10 min (Figure 6E). Consistently, HC-030031 restored thermal withdrawal latencies in the radiant heat assay (Figure 6F). To further confirm the involvement of TRPA1 in DMI-induced pain, we performed behavioral assays in Trpa1 knockout (Trpa1^−/−^) mice. Compared to vehicle-treated controls, Trpa1^−/−^ mice showed no significant change in mechanical thresholds following intraplantar injection of 250 μg DMI (Figures 6G,H). Collectively, these results strongly support that TRPA1 activation is essential for DMI-induced mechanical and thermal hypersensitivity in mice.

To further assess whether the desensitization of TRPA1 induced by repeated DMI exposure affects pain behavior, we performed intraplantar injections of DMI (25, 250 μg, or 1 mg) or vehicle for three consecutive days and monitored changes in mechanical withdrawal thresholds (Figure 6I). On the first day, mice receiving 250 μg or 1 mg DMI exhibited significantly reduced mechanical thresholds, indicative of mechanical hypersensitivity (Figure 6J). However, on the second and third days, the mechanical thresholds in all DMI-treated groups gradually returned to baseline and showed no significant difference compared to the vehicle group (Figure 6J). These findings support the hypothesis that repeated DMI exposure induces TRPA1 desensitization, which contributes to its delayed anti-nociceptive effects.

### 3.7 Repeated intraperitoneal injection of DMI does not affect baseline pain threshold and open-field behavior in mice

**FIGURE 7:**
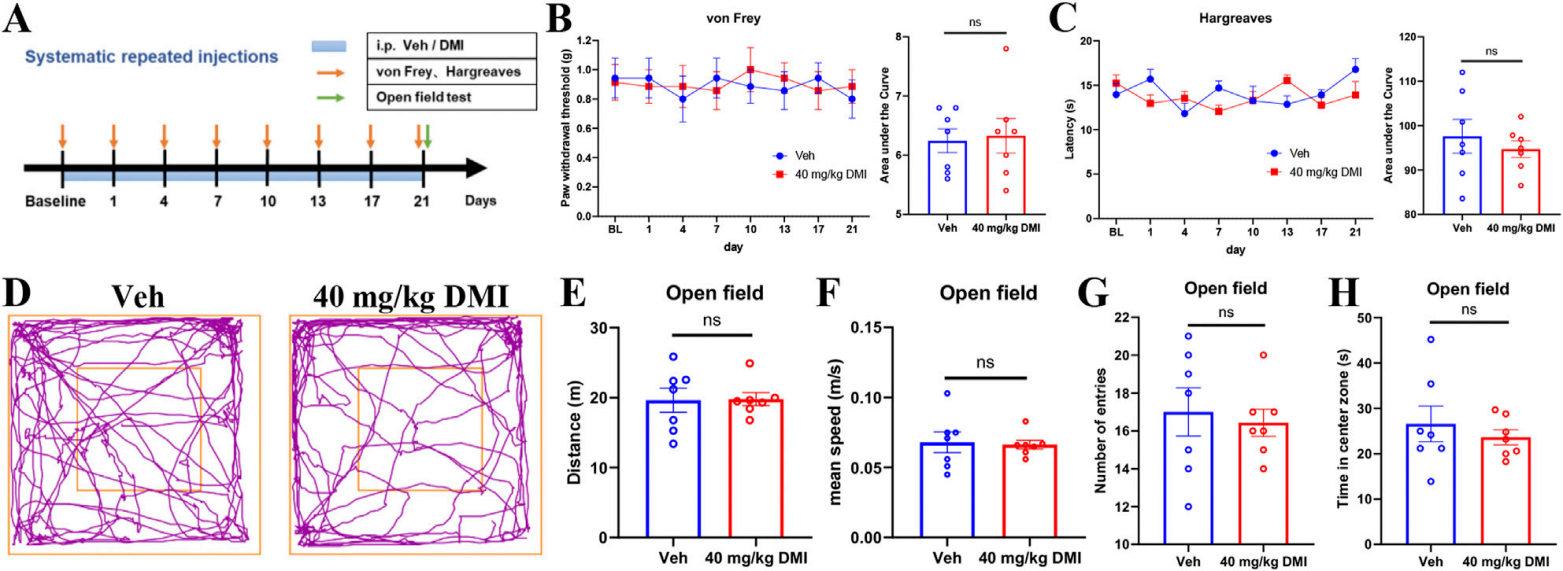
Repeated administration of DMI does not affect basal pain sensitivity and open-field behaviors in mice. **(A)** Timeline of repeated DMI administration. **(B)** Mechanical withdrawal thresholds of the hind paw and corresponding area under the curve (AUC) analysis following DMI treatment. **(C)** Paw withdrawal latency to radiant heat stimulation and corresponding AUC analysis. **(D)** Representative locomotor tracking plots in the open field test. **(E)** Total distance traveled in the open field over 5 min. **(F)** Average velocity during the 5-min open field test. **(G)** Number of entries into the central zone. **(H)** Time spent in the central zone. (n = 7 per group; respectively, two-way ANOVA or unpaired, two-tailed Student’s t-test).

Given our prior observations that repeated intraplantar administration of DMI may induce tactile allodynia desensitization, we further investigated the long-term effects of systemic DMI treatment on pain sensitivity and emotional behaviors. Mice were intraperitoneally injected with DMI (40 mg/kg) once daily for three consecutive weeks. Mechanical pain thresholds were assessed using von Frey filaments and thermal nociceptive thresholds were evaluated using a radiant heat assay every 3 days (Figure 7A). Throughout the 21-day observation period, no significant differences were observed in mechanical (Figure 7B) or thermal pain thresholds (Figure 7C) between the DMI-treated and vehicle-treated groups, suggesting that this dosing regimen does not induce adaptive changes in nociceptive behavior.

In addition, after 21 days of DMI administration, we conducted an open field test to evaluate spontaneous locomotor activity and anxiety-like behavior (Figure 7A). As demonstrated by representative traces (Figure 7D), there are no significant differences between DMI-treated and control mice in total distance traveled (Figure 7E), mean speed (Figure 7F), number of entries into the central zone (Figure 7G), or time spent in the center zone (Figure 7H). These findings indicate that chronic DMI administration does not affect locomotion or anxiety-like behaviors in mice.

### 3.8 Repeated intraperitoneal injection of DMI relieves pain behaviors and histological alterations in DSS-induced acute colitis in mice

**FIGURE 8:**
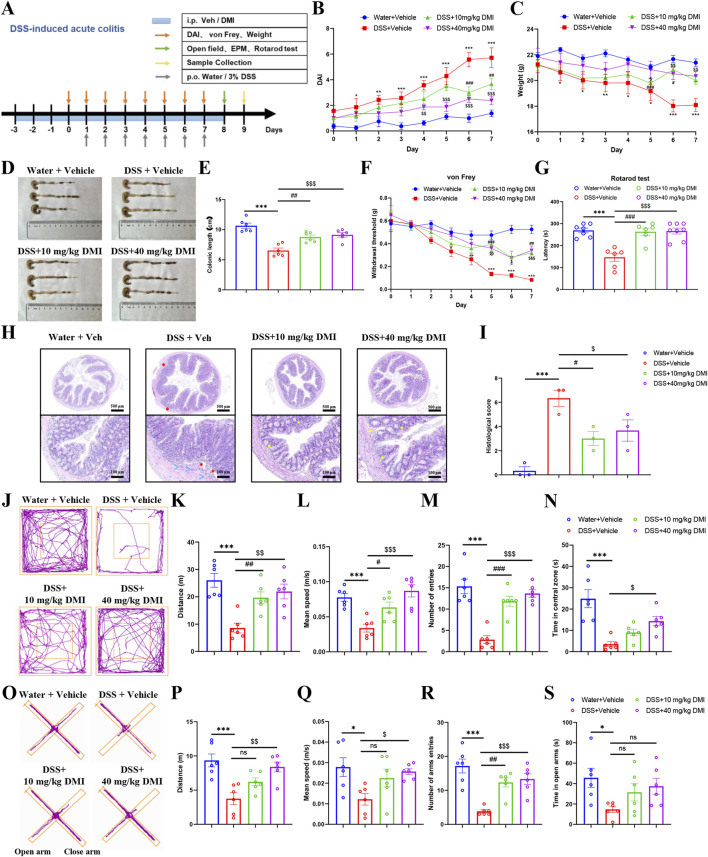
DMI alleviates DSS-induced acute colitis and associated pain, motor, and anxiety-like behaviors in mice. **(A)** Schematic diagram of the experimental timeline for DSS-induced colitis modeling, intraperitoneal DMI administration, and behavioral assessments. **(B)** Disease activity index (DAI) scores based on body weight loss, stool consistency, and fecal occult blood. **(C)** Body weight changes in mice across groups. **(D)** Representative images of colonic morphology. **(E)** Statistical analysis of colon length (n = 6 per group; ^*^ *P* < 0.05, ^**^ *P* < 0.01, ^***^ *P* < 0.001 vs. Water+ Vehicle; ^#^ *P* < 0.05, ^##^ *P* < 0.01, ^###^ *P* < 0.001 vs. DSS + Vehicle; ^$^ *P* < 0.05, ^$$^ *P* < 0.01, ^$$$^ *P* < 0.001 vs. DSS + Vehicle; respectively, one-way AVOVA following Bonferroni’s test or two-way AVOVA). **(F)** Abdominal mechanical hypersensitivity in DSS-induced colitis mice measured by von Frey filaments. **(G)** Latency to fall in the rotarod test (n = 7 per group; ^*^ *P* < 0.05, ^**^ *P* < 0.01, ^***^ *P* < 0.001 vs. Water+ Vehicle; ^##^ *P* < 0.01, ^###^ *P* < 0.001 vs. DSS +Vehicle; ^$^ *P* < 0.05, ^$$^ *P* < 0.01, ^$$$^ *P* < 0.001 vs. DSS + Vehicle; respectively, one-way AVOVA following Bonferroni’s test or two-way AVOVA). **(H)** Representative histological sections of mouse colon tissue stained with H&E (hematoxylin and eosin) in DSS-induced acute colitis model. Water + Veh group shows intact mucosal and epithelial architecture. DSS + Veh group displays extensive epithelial ulceration (red asterisks), crypt destruction, and inflammatory infiltration (blue arrows). DSS + DMI (10 mg/kg) shows partial crypt restoration and areas with increased goblet cells (yellow asterisks). DSS + DMI (40 mg/kg) reveals markedly improved mucosal architecture, crypt regeneration (yellow asterisks), and reduced inflammation. All sections were evaluated by a blinded pathologist using established scoring criteria. Upper panels: low-magnification images (scale bar = 500 μm), Lower panels: high-magnification views (scale bar = 100 μm). **(I)** Histological activity index (HAI) scores based on the number of ulcerated cross-sections, epithelial damage, and inflammatory cell infiltration (n = 3 per group; ^***^ *P* < 0.001 vs. Water + Vehicle; ^#^ *P* < 0.05, ^$^ *P* < 0.05 vs. DSS +Vehicle; one-way AVOVA following Bonferroni’s test). **(J)** Representative locomotor tracking plots in the open field test. **(K)** Total distance traveled during 5 min in the open field. **(L)** Average movement speed during the 5-min open field session. **(M)** Number of entries into the central zone. **(N)** Time spent in the central zone (n = 6 per group; ^***^ *P* < 0.001 vs. Water+ Vehicle; ^#^ *P* < 0.01, ^##^ *P* < 0.01, ^###^ *P* < 0.001 vs. DSS +Vehicle; ^$^ *P* < 0.05, ^$$^ *P* < 0.01, ^$$$^ *P* < 0.001 vs. DSS + Vehicle; one-way AVOVA following Bonferroni’s test). **(O)** Representative movement trajectories in the elevated plus maze test. **(P)** Total distance traveled during the 5-min test. **(Q)** Average velocity (m/s) during the test. **(R)** Number of entries into the open arms. **(S)** Time spent in the open arms (n = 6 per group; ^*^ *P* < 0.05, ^***^ *P* < 0.001 vs. Water + Vehicle; ^##^ *P* < 0.01 vs. DSS + Veh; ^$^ *P* < 0.05, ^$$^ *P* < 0.01, ^$$$^ *P* < 0.001 vs. DSS + Vehicle; one-way AVOVA following Bonferroni’s test).

The above findings indicate that repeated administration of DMI exerts anti-nociceptive effects through TRPA1 desensitization without inducing adaptive changes in pain behavior, motor function, or anxiety-like responses. We next explored the therapeutic potential of repeated DMI administration across models of acute and chronic pain. Inflammatory bowel disease (IBD) is a relapsing-remitting gastrointestinal disorder characterized by chronic inflammation and progressive structural and functional damage to the intestine, often accompanied by visceral pain (de Oliveira et al., 2023; Wu et al., 2023). To evaluate the efficacy of DMI in this context, we employed a dextran sulfate sodium (DSS)-induced acute colitis model. Mice were administered 3% DSS in drinking water for seven consecutive days (D1-D7) to induce colitis, followed by regular water. DMI was administered intraperitoneally at 10 or 40 mg/kg once daily, starting 3 days prior to DSS exposure and continuing throughout the experimental period. Disease severity was monitored daily by assessing changes in body weight, stool consistency, and rectal bleeding, which were integrated into a disease activity index (DAI). Visceral hypersensitivity was evaluated via abdominal mechanical pain threshold testing, and behavioral assessments-including open field test, elevated plus maze, and rotarod performance-were conducted on Day 8. On Day 9, colon tissues were harvested for gross morphological and histological analysis, including measurements of colon length and hematoxylin-eosin (H&E) staining (Figure 8A).

The results showed that DMI treatment significantly attenuated DSS-induced body weight loss, with the 40 mg/kg group demonstrating superior therapeutic efficacy (Figure 8C). DMI also markedly reduced DAI scores, indicating improvements in stool consistency and reduction in rectal bleeding (Figure 8B). Morphological assessments confirmed that DMI mitigated DSS-induced colon shortening, hyperemia, edema, and tissue fragility (Figures 8D,E).

Subsequently, we evaluated the development of visceral hypersensitivity using an abdominal mechanical pain threshold test. The results demonstrated that compared to control mice, DSS-treated mice exhibited a significant reduction in mechanical threshold beginning on day 3, indicating the development of inflammation-associated and post-inflammatory visceral hypersensitivity in response to noxious stimuli (Figure 8F). While DMI intervention significantly increased the mechanical threshold in DSS-treated mice, with the high-dose group (40 mg/kg) exhibiting the most pronounced analgesic effect between days 5 and 7 compared to the untreated model group (Figure 8F). To further assess the impact of DMI on motor function, we conducted the rotarod test. Following 3 days of training, motor coordination was evaluated on Day 8. The results indicated that DSS-treated mice exhibited a significantly reduced latency to fall in the rotarod test compared to control mice, indicating impaired motor coordination and performance. And DMI-treated mice displayed significantly improved performance compared to DSS-treated controls (Figure 8G), suggesting enhanced motor coordination. These findings indicate that DMI not only alleviates visceral pain hypersensitivity but also improves motor function in the DSS-induced acute colitis model.

Histological analysis revealed that colonic tissues from the DSS model group exhibited classical pathological features of inflammatory bowel disease (IBD), including multiple mucosal ulcerations with prominent edema, a marked reduction in mucus-secreting goblet cells, distorted and swollen crypt structures, and extensive infiltration of inflammatory cells-primarily neutrophils and mononuclear cells-within the mucosa and submucosa. The integrity of the epithelial barrier was also severely compromised (Figures 8H,I). These alterations confirmed the successful establishment of the colitis model. Notably, DMI treatment markedly ameliorated these pathological changes. Specifically, mice in the DMI-treated groups showed reduced ulcerated areas, diminished inflammatory cell infiltration, partial restoration of crypt architecture, and increased numbers of goblet cells. Of particular importance, the 40 mg/kg DMI group exhibited near-normal mucosal morphology, suggesting superior efficacy in promoting mucosal repair and controlling inflammation (Figures 8H,I). These findings indicate that DMI confers protective effects on the colonic mucosa in DSS-induced colitis by preserving epithelial integrity, facilitating tissue regeneration, and suppressing inflammatory responses.

In addition to visceral pain, patients with IBD might have psychological and emotional symptoms (Bakshi et al., 2021). To assess the effects of DMI on emotional behaviors in DSS-induced colitis mice, open field and elevated plus maze (EPM) tests were conducted on day 8 after model induction. As shown by the representative movement trajectories (Figures 8J,O), mice in the DSS model group exhibited significantly reduced total distance traveled (Figure 8K), mean speed (Figure 8L), frequency of center entries (Figure 8M), and time spent in the center area (Figure 8N) in the open field test, compared to control mice. DMI treatment significantly ameliorated anxiety-like behaviors in DSS mice, evidenced by increased center entry frequency in both DMI-treated groups (Figure 8M), along with improved total distance and average speed (Figures 8K,L). Notably, mice receiving 40 mg/kg DMI also showed a significant increase in center time compared to the DSS model group (Figure 8N). In the EPM test, DSS-treated mice demonstrated reduced total movement distance (Figure 8P), mean speed (Figure 8Q), number of open-arm entries (Figure 8R), and time spent in the open arms (Figure 8S) compared to controls. Treatment with 40 mg/kg DMI significantly increased number of open-arm entries (Figure 8R), total distance traveled (Figure 8P), and mean speed (Figure 8Q) relative to the DSS model group. The 10 mg/kg DMI group showed a significant increase only in the number of open-arm entries (Figure 8R), with no significant differences in open-arm duration in either DMI group relative to the DSS model group (Figure 8S). These results suggest that DMI administration alleviates DSS-induced anxiety-like emotional disturbances in mice.

Together, these findings demonstrate that DMI exerts significant therapeutic effects in the DSS-induced acute colitis model, alleviating both clinical symptoms and histopathological damage, while also improving pain-related and anxiety-like behavioral outcomes.

### 3.9 Repeated DMI administration alleviates pain behaviors in chemotherapy-induced neuropathic pain and bone cancer pain in mice

**FIGURE 9:**
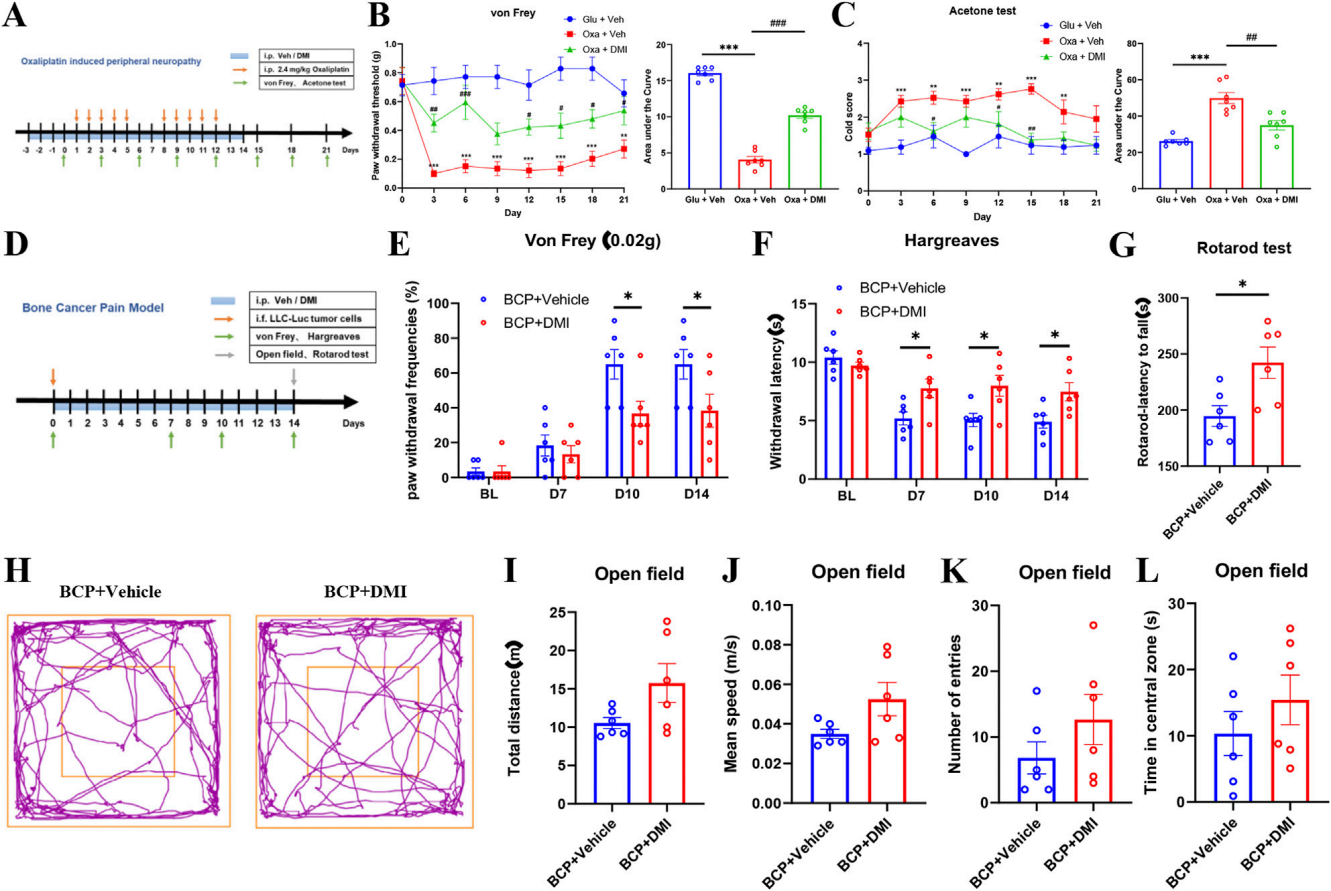
Systemic DMI administration alleviates pain behaviors in oxaliplatin-induced neuropathic pain and bone cancer pain in mice. **(A)** Schematic timeline of oxaliplatin-induced neuropathic pain model and behavioral testing. **(B)** Time course of mechanical pain thresholds and corresponding AUC analysis. **(C)** Acetone test scores for cold allodynia and AUC quantification (n = 7 per group; ^**^ *P* < 0.01, ^***^ *P* < 0.001, vs. Glucose + Vehicle group; ^#^ *P* < 0.05, ^##^ *P* < 0.01, ^###^ *P* < 0.001 vs. Oxaliplatin + Vehicle; respectively, two-way ANOVA or one-way AVOVA following Bonferroni’s test). **(D)** Schematic timeline of bone cancer pain model and behavioral testing. **(E)** Paw withdrawal frequencies (PWFs) in response to 0.02 g von Frey filament stimulation in bone cancer pain mice. **(F)** Paw withdrawal latency to radiant heat stimulation in bone cancer pain mice following DMI treatment. **(G)** Latency to fall in the rotarod test in bone cancer pain mice following DMI treatment. **(H)** Representative locomotor tracking plots in the open field test in bone cancer pain mice following DMI treatment. **(I)** Total distance traveled in the open field over 5 min. **(J)** Average velocity in the open field over 5 min. **(K)** Number of entries into the center zone. **(L)** Time spent in the center zone (n = 6 per group; ^*^ *P* < 0.05 vs. BCP + Vehicle; respectively, two-way ANOVA or unpaired, two-tailed Student’s t-test).

To further investigate the effect of DMI on other pain modalities, we established a chemotherapy-induced peripheral neuropathy (CIPN) model using a well-validated oxaliplatin administration protocol. Mice were intraperitoneally injected with oxaliplatin (2.4 mg/kg) following a “5-day injection + 2-day rest” cycle, repeated twice for a total of 10 doses. Pain-related behavioral phenotypes were dynamically assessed by measuring mechanical withdrawal thresholds and cold allodynia using von Frey filaments and acetone tests, respectively (Figure 9A). Behavioral analysis confirmed the development of mechanical and cold hypersensitivity in oxaliplatin-treated mice (Figures 9B,C), indicating successful model induction. To evaluate the therapeutic potential of DMI, a preventive dosing strategy was employed: mice received daily intraperitoneal injections of DMI (40 mg/kg) starting 3 days prior to the first oxaliplatin injection and continuing throughout the study (Figure 9A). The results demonstrated that DMI-treated mice exhibited significantly higher mechanical withdrawal thresholds and reduced cold allodynia scores compared to vehicle-treated CIPN controls (Figures 9B,C). These findings indicate that DMI effectively alleviates oxaliplatin-induced mechanical and cold hypersensitivity, supporting its potential as a therapeutic candidate for chemotherapy-induced neuropathic pain.

In addition, we evaluated the effects of DMI in a cancer-related pain model. Bone cancer pain was induced by intrafemoral injection of 2 μL LLC-Luc tumor cell suspension (2 × 10^5^ cells) into the left hind limb of mice. DMI treatment was initiated on day 0 of tumor inoculation with daily intraperitoneal administration of 40 mg/kg DMI until the end of the study. Behavioral assessments were conducted on days 0, 7, 10, and 14 post-inoculations, including mechanical hypersensitivity (von Frey test) and thermal hyperalgesia (Hargreaves test). On day 14, motor coordination and anxiety-like behaviors were further evaluated using the rotarod test and open field test, respectively (Figure 9D). In the mechanical sensitivity test, DMI-treated mice exhibited significantly elevated pain thresholds compared to vehicle-treated tumor-bearing mice on days 10 and 14 (Figure 9E). In the Hargreaves test, DMI treatment significantly prolonged paw withdrawal latency on days 7, 10, and 14 (Figure 9F). Rotarod analysis revealed improved motor coordination in the DMI group compared to the cancer pain model group (Figure 9G). In the open field test, representative movement trajectories (Figure 9H) showed that DMI-treated mice tended to exhibit increased total travel distance (Figure 9I), mean speed (Figure 9J), center entries (Figure 9K), and time spent in the center area (Figure 9L) compared to vehicle-treated cancer model mice; however, these differences were not statistically significant. Collectively, these results suggest that although DMI did not significantly alleviate anxiety-like behavior in the bone cancer pain model, it significantly improved mechanical and thermal pain thresholds as well as motor performance in tumor-bearing mice.

### 3.10 Repeated DMI administration alleviates acute and chronic inflammatory pain in mice

**FIGURE 10:**
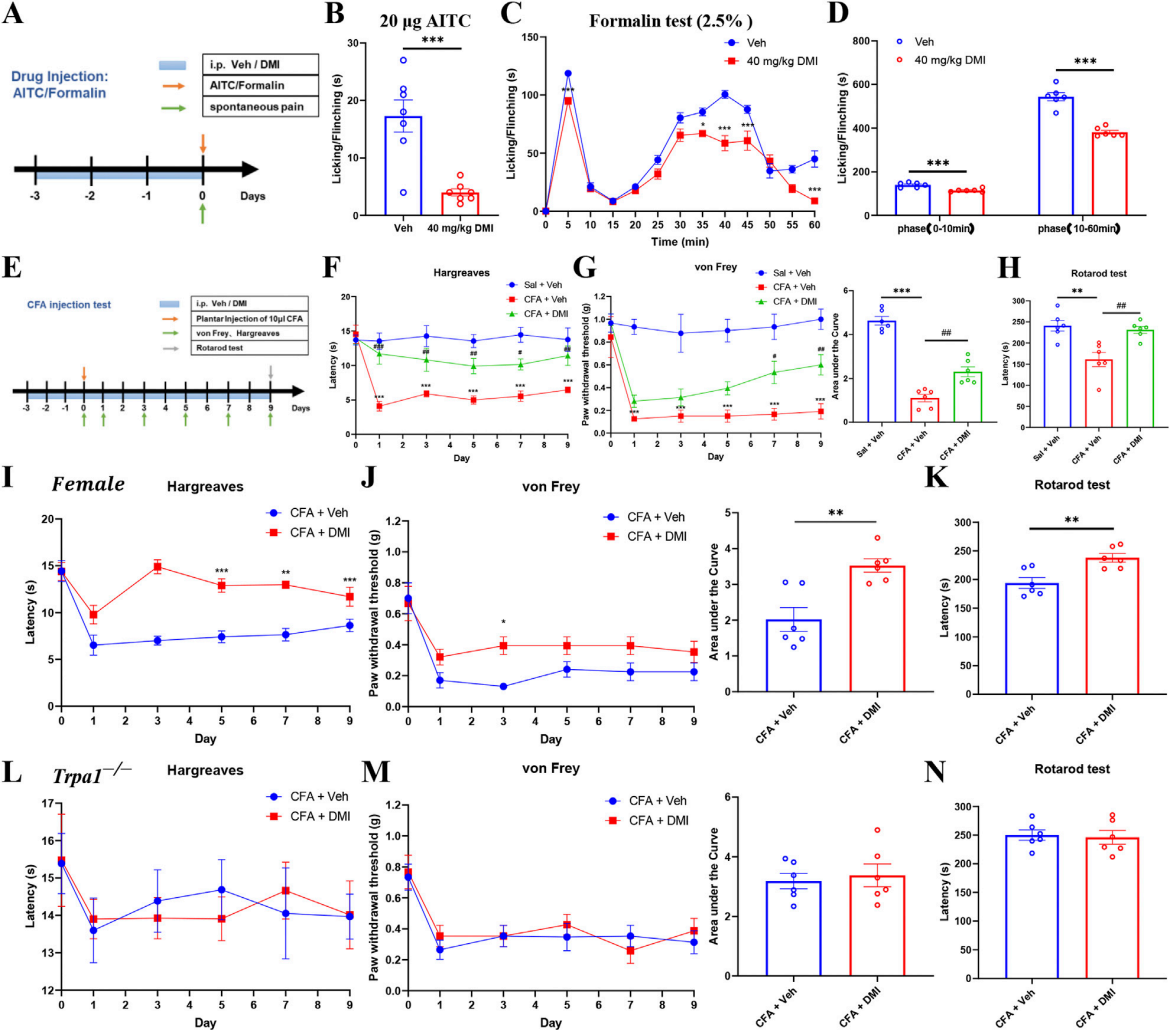
Systemic DMI administration alleviates pain behaviors in formalin-, AITC-, and CFA-induced pain in mice. **(A)** Schematic diagram of experimental procedures for plantar injection of AITC or formalin and subsequent behavioral assessments. **(B)** Cumulative time of spontaneous pain behaviors in the AITC model following repeated DMI administration. **(C)** Time course of paw licking or lifting behaviors every 5 min within 1 h after formalin injection. **(D)** Quantification of phase I and phase II paw licking/lifting durations in the formalin model (n = 6 per group; *P < 0.05, ***P < 0.001, vs. Vehicle group; respectively, two-way ANOVA or unpaired, two-tailed Student’s t-test). **(E)** Schematic diagram of experimental procedures for the CFA-induced inflammatory pain model and behavioral testing. **(F)** Paw withdrawal latency to radiant heat stimulation in CFA-treated mice following DMI treatment. **(G)** Mechanical pain thresholds measured by von Frey test and corresponding area under the curve (AUC) analysis in CFA-treated mice following DMI treatment. **(H)** Latency to fall in the rotarod test in CFA-treated mice following DMI treatment (n = 6 per group; ^**^ *P* < 0.01, ^***^ *P* < 0.001, vs. Saline + Vehicle group; ^#^ *P* < 0.05, ^##^ *P* < 0.01, ^###^ *P* < 0.001 vs. CFA + Vehicle; respectively, one-way AVOVA following Bonferroni’s test, two-way AVOVA, or unpaired, two-tailed Student’s t-test). **(I)** Paw withdrawal latency to radiant heat stimulation in CFA-treated female mice following DMI treatment. **(J)** Mechanical pain thresholds measured by von Frey test and corresponding area under the curve (AUC) analysis in CFA-treated female mice following DMI treatment. **(K)** Latency to fall in the rotarod test in CFA-treated female mice following DMI treatment (n = 6 per group; ^*^ *P* < 0.05, ^**^ *P* < 0.01, ^***^ *P* < 0.001, vs. Female-CFA + Vehicle group; two-way AVOVA, or unpaired, two-tailed Student’s t-test). **(L)** Paw withdrawal latency to radiant heat stimulation in CFA-treated Trpa1−/− mice following DMI treatment. **(M)** Mechanical pain thresholds measured by von Frey test and corresponding area under the curve (AUC) analysis in CFA-treated Trpa1−/− mice following DMI treatment. **(N)** Latency to fall in the rotarod test in CFA-treated Trpa1−/− mice following DMI treatment (n = 6 per group; two-way AVOVA, or unpaired, two-tailed Student’s t-test).

Local exposure to TRPA1 agonists, even at sub-nociceptive doses, is known to elicit immediate nociceptive responses lasting only a few minutes. AITC, a well-established TRPA1 agonist, induces pain-like behaviors in naïve animals in a dose-dependent manner (Fialho et al., 2023). The formalin test, a widely accepted model of inflammatory pain, allows for the quantification of biphasic nocifensive behavior in rodents and is considered a robust tool in preclinical analgesic drug evaluation (López-Cano et al., 2017). To assess the potential modulatory effect of DMI on acute nociceptive behavior, we employed two classical models: an AITC-induced acute chemical pain model (TRPA1-dependent) and a formalin-induced inflammatory pain model. Mice were pretreated with DMI (40 mg/kg, i.p.) once daily for three consecutive days, followed by intraplantar injection of AITC (20 μg) or 2.5% formalin (10 μL) into the left hind paw (Figure 10A). Behavioral analysis revealed that DMI pretreatment significantly reduced spontaneous nocifensive responses within the first 10 min after AITC injection compared to vehicle-treated model mice (Figure 9B). In the formalin test, DMI pretreatment also exerted a significant analgesic effect during both the first phase (0–10 min) and second phase (10–60 min) of pain behavior (Figures 9C,D), as evidenced by a marked reduction in cumulative paw licking and flinching time relative to controls (Figures 10C,D).

Complete Freund’s adjuvant (CFA) is widely used to induce chronic inflammatory pain in rodent models, characterized by mechanical and thermal hypersensitivity. Chronic inflammation leads to the release of pro-inflammatory mediators from the site of injury and surrounding tissues, lowering the pain threshold and amplifying nociceptive responses (Kiguchi et al., 2009). To evaluate the potential modulatory effects of DMI on chronic inflammatory pain, we established a CFA-induced inflammatory pain model. Mice were pretreated with DMI (40 mg/kg, i.p.) once daily for three consecutive days prior to model induction. On the next day, 10 μL CFA (1 mg/mL) was injected subcutaneously into the plantar surface of the left hind paw. DMI administration was continued daily until the end of the experiment. Mechanical allodynia (von Frey test) and thermal hyperalgesia (Hargreaves test) were assessed on days 1, 3, 5, 7, and 9 after CFA injection. On day 9, motor coordination was evaluated using the rotarod test (Figure 10E). Behavioral results demonstrated that mice in the CFA group developed significant thermal hypersensitivity from day 1, as evidenced by decreased paw withdrawal latency. DMI treatment significantly increased withdrawal latency compared to the CFA group from the first day onward (Figure 10F). Similarly, in mechanical sensitivity tests, CFA-injected mice exhibited a persistent decrease in paw withdrawal threshold starting from day 1, while DMI-treated mice showed a significant analgesic effect on days 7 and 9. Quantification of the area under the curve (AUC) revealed a statistically significant analgesic effect in the DMI group (Figure 10G). Furthermore, in the rotarod test, CFA-treated mice exhibited impaired motor performance compared to the control group, whereas DMI treatment significantly improved retention time on the rotating rod, indicating amelioration of pain-associated motor deficits (Figure 10H). Collectively, these results confirm that DMI exerts a robust analgesic effect in a chronic inflammatory pain model by alleviating both thermal and mechanical hypersensitivity and improving pain-related motor dysfunction.

To evaluate whether the analgesic effect of DMI exhibits sex-specific differences, we established the CFA-induced inflammatory pain model in female mice. Behavioral assays demonstrated that, compared to vehicle-treated controls, DMI administration significantly increased paw withdrawal latency in thermal sensitivity tests starting from day 5 post-injection (Figure 10I). Similarly, in the mechanical sensitivity test, DMI-treated mice showed a significant antinociceptive effect in day 3. Quantification of the AUC confirmed a statistically significant alleviation in pain responses in the DMI group (Figure 10J). Furthermore, in the rotarod test, DMI-treated mice exhibited improved performance, with significantly prolonged latency to fall, indicating an amelioration of pain-related motor impairments (Figure 10K). These results are consistent with findings obtained in male mice and suggest that the analgesic effect of DMI mediated via TRPA1 desensitization is not sex-specific under these conditions.

To further confirm that DMI’s analgesic action is indeed mediated by TRPA1 desensitization, we employed Trpa1 knockout (Trpa1^−/−^) mice in the CFA pain model. Notably, DMI treatment failed to produce additional analgesic effects in Trpa1^−/−^ mice, as reflected by no significant improvements in thermal sensitivity (Figure 10L), mechanical sensitivity (Figure 10M), or motor performance (Figure 10N) compared to vehicle controls. These findings indicate that the absence of TRPA1 abolishes the analgesic efficacy of DMI, reinforcing the conclusion that DMI exerts its antinociceptive effects predominantly through TRPA1 desensitization.

## 4 Discussion

The present study elucidates a novel mechanism of action by which dimethyl itaconate (DMI), a derivative of the endogenous metabolite itaconate, directly activates the transient receptor potential ankyrin 1 (TRPA1) channel, triggers functional desensitization, and subsequently attenuates pain behaviors in a range of acute and chronic pain models. Our findings provide a comprehensive understanding of the molecular interactions between DMI and TRPA1, highlight the functional consequences of repeated TRPA1 activation, and propose a new pharmacological strategy for pain management through TRPA1 desensitization rather than inhibition.

TRPA1 is a non-selective cation channel predominantly expressed in nociceptive sensory neurons and is known to respond to a wide range of noxious stimuli, including electrophiles, oxidants, and environmental irritants (Moparthi et al., 2014). Previous studies have implicated TRPA1 in the pathogenesis of inflammatory (McMahon and Wood, 2006), neuropathic (De Logu et al., 2017; Li et al., 2020), and visceral pain (Meseguer et al., 2014). In our work, the calcium imaging in hTRPA1-transfected HEK293T cells demonstrated that DMI acutely and robustly activates TRPA1 in a concentration-dependent manner, an effect that was significantly inhibited by the selective TRPA1 antagonist HC-030031. Notably, DMI failed to evoke calcium responses in either mock-transfected or TRPV1-overexpressing HEK293T cells, indicating target specificity. The EC_50_ value of DMI in hTRPA1-expressing cells was approximately 89.42 μM, suggesting that DMI is a moderately potent TRPA1 agonist. Additionally, calcium imaging in primary mouse dorsal root ganglion (DRG) neurons revealed that DMI also activated endogenous TRPA1, and this response was absent in DRG neurons derived from Trpa1 knockout mice but preserved in Trpv1-deficient cells, further confirming TRPA1 selectivity. These findings collectively suggest that DMI selectively activates TRPA1 both in recombinant and native neuronal systems. However, calcium imaging approaches have inherent limitations. The EC_50_ value obtained from hTRPA1-transfected cells provides only an approximate measure of DMI potency, as intracellular redox buffers such as glutathione can quench reactive DMI, thereby limiting effective channel activation *in vivo* (Lang and Siddique, 2024; Ma et al., 2025). More precise evaluation of TRPA1 activation could be achieved using inside-out patch clamp techniques, which minimize cytosolic buffering and allow direct assessment of channel gating. Therefore, future studies should incorporate these methodological refinements to enhance our understanding about the molecular mechanism of DMI acting on TRPA1 channel.

Previous studies have indicated that TRPA1 is a redox-sensitive ion channel capable of detecting reactive electrophilic species, many of which form covalent adducts with conserved cysteine residues located in the N-terminal domain (Macpherson et al., 2007). Among these residues, Cys621 has been repeatedly shown to be indispensable for covalent agonist binding (Bahia et al., 2016). Our molecular docking and covalent docking simulation revealed that DMI forms a stable covalent interaction with Cys621, while also engaging multiple hydrogen bonding and hydrophobic interactions within the ligand-binding pocket. Importantly, site-directed mutagenesis of Cys621 abolished DMI-induced calcium signals, strongly supporting the role of this residue in the channel activation mechanism. Furthermore, DMI-induced responses were significantly attenuated by the selective TRPA1 antagonist HC-030031, providing additional pharmacological validation of TRPA1 as the functional target. Given DMI’s electrophilic α, β-unsaturated carbonyl structure (Mills et al., 2018), it likely acts as an electrophile, forming covalent adducts with cysteine residues. The combined chemical, functional, pharmacological, and mutagenesis evidence provides compelling support for TRPA1 as the important molecular target of DMI, although other potential targets of DMI were not excluded in the present study.

An increasing body of evidence has demonstrated that TRPA1 agonists can serve as an effective strategy to pain management by desensitizing TRPA1 currents (Bressan et al., 2016). One of the most notable findings from our study is the observation that repeated application of DMI leads to TRPA1 desensitization, characterized by a progressive reduction in calcium responses upon subsequent exposures. This phenomenon was observed in both heterologous hTRPA1-expressing cells and in native DRG neurons, which mirrors the tachyphylaxis observed with many TRP channel agonists and supports the hypothesis that DMI not only serves as an activator but also induces functional desensitization. A critical aspect of TRPA1 desensitization by DMI pertains to whether this effect is limited to homologous desensitization, where only DMI loses its efficacy upon repeated exposure, or whether it extends to heterologous desensitization, where other TRPA1 agonists such as AITC are also unable to activate the channel following DMI pretreatment. While our study demonstrated that repeated application of DMI leads to progressively reduced intracellular calcium responses in hTRPA1-expressing cells and DRG neurons, further experiments are necessary to determine whether this desensitization prevents subsequent activation by structurally unrelated agonists. Given the covalent nature of DMI binding to Cys621, it is plausible that its interaction induces a conformational or steric hindrance that precludes channel gating even by other electrophilic agonists. This would suggest a broader desensitization profile and could provide a therapeutic advantage by rendering TRPA1 globally less responsive under pathological conditions characterized by excessive activation. Alternatively, if DMI-induced desensitization is agonist-specific, it may point toward a distinct conformational adaptation or internalization pathway that only affects DMI-triggered activation. Dissecting the extent of TRPA1 desensitization and the persistence of this refractory state following DMI exposure will be essential for assessing its translational potential and determining optimal dosing regimens for chronic pain management.

Nonetheless, behaviorally, repeated intraplantar injections of DMI produced an initial reduction in mechanical threshold, followed by a diminished pain response upon subsequent administration, further supporting TRPA1 desensitization *in vivo*. More importantly, systemic administration of DMI exhibited broad analgesic effects across several models of acute and chronic pain, including DSS-induced colitis, CFA-induced inflammatory pain, AITC/formalin-induced chemical nociception, oxaliplatin-induced neuropathic pain, and bone cancer pain. These findings suggest that TRPA1 desensitization by DMI contributes to a generalized anti-nociceptive effect. Notably, an important aspect of our study is the demonstration that chronic DMI administration does not alter basal pain thresholds, locomotor activity, or emotional behavior in naïve mice. Neither acute nor chronic DMI treatment produced hyperlocomotion, sedation, or anxiogenic effects, indicating that the analgesic effects are not confounded by general behavioral suppression. Furthermore, the lack of tolerance development over repeated dosing supports the utility of DMI as a desensitizing agent that retains efficacy across multiple administrations. However, most current *in vivo* studies have employed intraperitoneal (i.p.) administration of DMI to ensure consistent systemic exposure and to minimize variability caused by first-pass metabolism. Given the potential translational value, the anti-nociception by oral administration of DMI warrants further investigation.

To further explore the functional relevance of TRPA1 desensitization by DMI, we employed multiple models of pain. In the AITC-induced acute chemical pain model, DMI pre-treatment significantly reduced spontaneous nocifensive behavior, consistent with TRPA1 inhibition. Additionally, in the formalin test, DMI reduced both the early (neurogenic) and late (inflammatory) phases of pain behavior. These data are consistent with TRPA1’s dual role in acute nociception and neurogenic inflammation. In the CFA-induced chronic inflammatory pain model, systemic administration of DMI effectively reduced both mechanical and thermal hyperalgesia. TRPA1 is known to be upregulated in inflamed tissues and contributes to peripheral and central sensitization through the release of neuropeptides such as CGRP and substance P (Bautista et al., 2013; Shibata and Tang, 2021; Landini et al., 2022; Yao et al., 2023). DMI, as a membrane-permeable derivative of itaconate, activates the Nrf2 antioxidant pathway and inhibits NF-κB-driven transcription, thereby reducing oxidative stress and pro-inflammatory cytokine release in both peripheral immune cells and spinal glial populations (Kuo et al., 2020; Ren et al., 2022). The anti-inflammatory and immunomodulatory properties of DMI may represent an important alternative mechanism contributing to analgesic effects of DMI, besides its modulatory effects on TRPA1 activity. This immunomodulatory may synergize with TRPA1 modulation, leading to pronounced anti-nociceptive effect of DMI across various pain models.

Inflammatory bowel disease (IBD) is frequently associated with visceral hypersensitivity and emotional comorbidities such as anxiety (Wu et al., 2023). In our DSS-induced colitis model, DMI alleviated disease activity index (DAI), preserved colonic architecture, and significantly elevated mechanical thresholds in the abdomen. These data indicate that DMI not only modulates immune and epithelial responses in the gut but also improves visceral pain, likely through modulation of peripheral sensory neurons expressing TRPA1. Consistent with this, DMI-treated animals exhibited improved performance in open field and elevated plus maze tests, reflecting reduced anxiety-like behaviors, which are commonly exacerbated by visceral inflammation.

Chemotherapy-induced peripheral neuropathy (CIPN) remains a debilitating adverse effect of platinum-based agents like oxaliplatin (Flatters et al., 2017). Our study shows that DMI prevents the development of mechanical allodynia and cold hyperalgesia in a CIPN model, suggesting a neuroprotective or desensitizing action at TRPA1-expressing fibers. Previous research has established TRPA1 as a critical contributor to oxaliplatin-induced pain via ROS and mitochondrial dysfunction (Nassini et al., 2011; Miyake et al., 2016; Leo et al., 2020). DMI, known for its anti-inflammatory and NRF2-activating properties (Rahimi et al., 2024; Wang et al., 2024), may mitigate such mechanisms while concurrently desensitizing TRPA1. We also demonstrated that DMI mitigates mechanical and thermal hyperalgesia in a mouse model of bone cancer pain. Although it did not significantly improve anxiety-like behavior in this context, DMI treatment ameliorated motor coordination deficits and extended paw withdrawal latency. Of note, our preliminary data showed that DMI did not induce cytotoxic effects on LLC cells (data not shown). Given that DMI may have complex effects on cancer cells, cancer micro-environment, and bone cancer pain, the possible application of DMI in the context of oncology may need to be further studied.

Taken together, our data support a model in which DMI covalently activates and subsequently desensitizes TRPA1, leading to suppression of nociceptive signaling. The combination of molecular, cellular, and behavioral evidence provides strong support for targeting TRPA1 desensitization as a strategy for pain control. Unlike TRPA1 antagonists, which may completely block physiological and protective pain signaling, partial agonist-induced desensitization allows a nuanced modulation of channel activity. This approach may offer advantages in terms of efficacy and safety, particularly in chronic or complex pain conditions. However, several questions remain. First, the precise intracellular pathways leading to TRPA1 desensitization by DMI remain to be elucidated. Second, the long-term safety of sustained TRPA1 desensitization has not been fully explored. While our administration did not produce overt side effects, longer treatment durations may reveal compensatory changes or off-target effects. In addition, whether DMI can cross the blood-brain barrier and modulate central TRPA1 remains unknown. Further pharmacokinetic and pharmacodynamic studies on DMI are warranted.

In conclusion, we have identified DMI as a novel TRPA1 agonist capable of inducing rapid functional desensitization and providing analgesia in multiple pain mouse models. These findings improve our understanding of pharmacological effects of DMI and support the further development of DMI or its derivatives as candidate therapeutics for management of acute and chronic pain.

## Data Availability

The original contributions presented in the study are included in the article/supplementary material, further inquiries can be directed to the corresponding authors.
